# Truncated DAPK Variants Restore Tumor Suppressor Activity and Synergize with Standard Therapies in High-Grade Serous Ovarian Cancer

**DOI:** 10.3390/cancers17121910

**Published:** 2025-06-08

**Authors:** Monika Raab, Khayal Gasimli, Balázs Győrffy, Samuel Peña-Llopis, Sven Becker, Mourad Sanhaji, Klaus Strebhardt

**Affiliations:** 1 Department of Gynecology, Medical School, Goethe-University, 60596 Frankfurt am Main, Germany; mraab@med.uni-frankfurt.de (M.R.); khayal.gasimli@unimedizin-ffm.de (K.G.); sven.becker@ukffm.de (S.B.); strebhardt@em.uni.frankfurt.de (K.S.); 2 Department of Bioinformatics, Semmelweis University, 1094 Budapest, Hungary; gyorffy.balazs@yahoo.com; 3 Department of Biophysics, Medical School, University of Pecs, 7624 Pecs, Hungary; 4 HUN-REN TTK Cancer Biomarker Research Group, 1117 Budapest, Hungary; 5 Translational Genomics, Department of Ophthalmology, University Hospital Essen, 45147 Essen, Germany; samuel.pena-llopis@uk-essen.de; 6 German Cancer Consortium (DKTK) at the University Hospital Essen and German Cancer Research Center (DKFZ), 69120 Heidelberg, Germany; 7 German Cancer Research Center (DKFZ), 69120 Heidelberg, Germany

**Keywords:** death-associated protein kinase 1 (DAPK1), p53, tumor suppressor, high-grade serous ovarian cancer (HGSOC), MDM2, mammalian vector-based DAPK1 expression, DAPK1 IVT-mRNA, drug resistance

## Abstract

Background/Objectives: Death-associated protein kinase 1 (DAPK1) is a serine/threonine kinase that plays a crucial role in cancer by regulating apoptosis through interactions with TP53. Aberrant expression of DAPK1 was shown in certain types of human cancer contributing to tumor progression and chemoresistance. This study aimed to investigate the role of DAPK1 in high-grade serous ovarian cancer (HGSOC) and to evaluate the therapeutic potential of restoring its kinase activity, including the use of truncated DAPK1 variants, to overcome chemoresistance and enhance tumor suppression. Methods: Gene expression analysis was performed on ovarian cancer tissues compared to benign controls to assess DAPK1 downregulation and its epigenetic regulation. Prognostic relevance was evaluated in a cohort of 1436 HGSOC patient samples. Functional restoration of DAPK1 was conducted in HGSOC cell lines and patient-derived primary tumor cells using vector-based expression or in vitro-transcribed (IVT) DAPK1 mRNA, including the application of truncated DAPK1 (ΔDAPK1) forms. To assess apoptosis, Caspase activation assays, 2D-colony formation assays, and cell survival assays were performed. To analyze the reactivation of DAPK1 downstream signaling, phosphorylation of p53 at Ser20 and the expression of p53 target proteins were examined. Chemosensitivity to Paclitaxel and Cisplatin was quantified by changes in IC_50_ values. Results: DAPK1 expression was significantly downregulated in ovarian cancer compared to benign tissue, correlating with epigenetic silencing, and showed prognostic value in early-stage HGSOC. Restoration of DAPK1 activity, including ΔDAPK1 variants, led to phosphorylation of p53 Ser20, increased expression of p53 target proteins, and Caspase-dependent apoptosis. Reactivation of DAPK1 sensitized both established HGSOC cell lines and patient-derived ascites cells to Paclitaxel and Cisplatin. These effects occurred through both p53-dependent and p53-independent pathways, enabling robust tumor suppression even in p53-mutant contexts. Conclusions: Reactivation of DAPK1, particularly through truncated variants, represents a promising therapeutic strategy to overcome chemoresistance in HGSOC. The dual mechanisms of tumor suppression provide a strong rationale for developing DAPK1-based therapies to enhance the efficacy of standard chemotherapy, especially in patients with chemoresistant or p53-deficient tumors. Future work should focus on optimizing delivery approaches for DAPK1 variants and assessing their synergistic potential with emerging targeted treatments in clinical settings.

## Simple Summary

Chemoresistance remains a major therapeutic challenge in high-grade serous ovarian cancer (HGSOC). Our study demonstrates that the functional restoration of death-associated protein kinase 1 (DAPK1), a novel tumor suppressor in HGSOC, induces potent tumor-specific cytotoxicity and inverts chemoresistance. Using molecular and preclinical analyses, we showed that full-length and truncated DAPK1 variants efficiently restore apoptotic signaling pathways and significantly enhance platinum-based and taxane treatment sensitivity. Therapeutic efficacy was validated across different model systems, including ovarian cancer cell lines and patient-derived organoids. Remarkably, the truncated DAPK1 constructs maintain strong apoptotic activity despite structural modifications, highlighting clinical potential. These findings suggest DAPK1 reactivation as a viable strategy to overcome treatment resistance in HGSOC, with the potential for translation into combination therapy.

## 1. Introduction

High-grade serous ovarian cancer (HGSOC) is the most common and aggressive form of ovarian cancer [1,2]. About 19,000 new cases of ovarian cancer are diagnosed annually in the US, of which ~70% (approximately 13,300) are this subtype. Across Europe, roughly 65,000 women are diagnosed with ovarian cancer each year; applying the same percentage range (70%) suggests roughly 45,500 annual cases of this malignancy. Similarly to many cancers, the risk increases with age, particularly in post-menopausal women. The 5-year survival rate for HGSOC varies depending on factors such as stage at diagnosis and patient health. For advanced cases, survival rates typically range from 20% to 40%. Early detection and advances in treatment options can improve patient survival and quality of life. However, the symptoms of HGSOC are often vague and non-specific, such as bloating, pelvic pain, and abdominal discomfort, leading to a late diagnosis when the cancer is already advanced. Specific biomarkers for HGSOC remain rare [3]. This ovarian cancer subtype is also notorious for its high recurrence rates; even after a successful initial treatment, it often returns with diminishing therapeutic efficacy over time.

Comprehensive research using pathway network tools to evaluate somatic copy-number alterations may help to guide the therapy of this malignancy [4]. Additionally, studies on genetic factors have identified a strong association between this cancer and mutations in the *BRCA1* and *BRCA2* genes, which are also implicated in breast cancer [5]. Women with these genetic mutations face a significantly higher risk of developing HGSOC [5]. A family history of ovarian, breast, or hereditary nonpolyposis colorectal cancer (Lynch syndrome) further increases susceptibility to high-grade serous ovarian carcinoma. Critically, *p53* mutations are present in over 96% of cases, abolishing its tumor-suppressive function [6]. These alterations disrupt cell cycle regulation, impair DNA repair, and inhibit apoptosis, driving aggressive tumor progression and genomic instability. Mutant *p53* also contributes to chemoresistance, diminishing the efficacy of conventional therapies [7,8]. Consequently, patients harboring these mutations face poorer prognoses and reduced survival rates. Current research prioritizes strategies to either restore wild-type *p53* activity or target its downstream pathways, with the goal of enhancing the therapeutic response and enabling personalized treatment approaches.

Death-associated protein kinase 1 (DAPK1) is a serine/threonine kinase involved in the regulation of apoptosis [9]. In certain cell types like neurons, the interaction of DAPK1 and p53 promotes cell death in response to stress [10]. Furthermore, DAPK1 increases the transcription of *ARF (p14ARF)*, a tumor suppressor protein that stabilizes p53 by inhibiting MDM-2-mediated ubiquitination [11]. DAPK1 enhances p53’s pro-apoptotic functions, contributing to the prevention of tumorigenesis by eliminating damaged cells. This kinase belongs to the family of calcium/calmodulin (CaM)-regulated serine/threonine protein kinases. The *DAPK* gene is evolutionarily conserved across mammals, chordates, and invertebrates such as *C. elegans*, and is located on chromosome 9q21.23 in humans. The N-terminal end of DAPK1 contains a conventional kinase domain and a Ca^2+^/CaM autoregulatory region that, in the absence of CaM, binds the catalytic cleft to inhibit enzymatic activity and block substrate access [12]. Upon CaM binding, the autoregulation domain dissociates from the catalytic region, enabling substrate phosphorylation. The autophosphorylation of the CaM autoregulatory domain at pSer308 reduces its affinity for CaM, thus inhibiting DAPK1’s enzymatic activity. The death domain, ankyrin repeats (AR), the Ras of complex protein (ROC), C-terminal of ROC (COR) domain, and the Ca^2+^/CaM autoregulatory domain follow towards the carboxyterminal end of DAPK1. Ankyrin repeats mediate protein–protein interaction and facilitate the degradation of DAPK1. The ROC-COR domain promotes GTP hydrolysis, indicating DAPK1’s role as GTPase and its involvement in cytoskeletal localization. Additionally, the ROC and kinase domains facilitate homodimerization. Many apoptosis-promoting proteins contain a protein–protein interaction domain similar to the death domain at the carboxyterminal end of DAPK1. DAPK1 has been linked to several critical pathways that result in autophagy and apoptosis [9]. By modulating these pathways, *DAPK1* acts as a tumor suppressor inhibiting tumor growth and metastasis.

Emerging evidence suggests that DAPK1 downregulation may contribute to disease progression, particularly in early-stage high-grade serous ovarian cancer, though the mechanistic links remain poorly understood. While DAPK1’s role in apoptosis is well established, its therapeutic potential in HGSOC, especially in the context of mutant p53, has not been fully explored. This gap is clinically relevant, as mutant p53 drives chemoresistance and poor outcomes. Here, we investigate whether restoring DAPK1 activity can overcome *p53*-related therapeutic resistance using both vector-based and mRNA-driven approaches. Our study aims to clarify how DAPK1 reactivation influences *p53* signaling and its tumor-suppressive mechanisms, offering insights into targeted therapies for this aggressive subtype of ovarian cancer.

## 2. Materials and Methods

### 2.1. Survival Analysis

We searched for ovarian cancer cohorts in the NCBI Gene Expression Omnibus (GEO, https://www.ncbi.nlm.nih.gov/geo/, accessed on 1 August 2024) and the Genomic Data Commons Data Portal (GDC, https://portal.gdc.cancer.gov/, accessed on 1 August 2024). Only samples with transcriptome-level data from at least ten patients were included. To ensure consistency in gene expression detection, we limited our search to tumor samples analyzed using three in situ oligonucleotide array platforms with identical probe sequences, including the GEO platforms GPL96, GPL571, and GPL570.

The oligonucleotide gene array files were MAS5 normalized, followed by a second scaling normalization to set the mean expression in each array to 1000, using only probes from the GPL96 platform. Quality control included checks for background intensity, noise, percentage of present calls, bioBCD spikes, and the GAPDH/ACTB 3 to 5 ratio. JetSet was used to select the most reliable probe set for each gene.

Cox proportional hazards regression was used to compute differential survival. Univariate analysis was performed for DAPK1 using all available cutoff values between the lower and upper quartiles of expression. The Benjamini–Hochberg False Discovery Rate method was applied to correct for multiple hypothesis testing. The most significant cutoff value was selected for the final analysis. Kaplan–Meier plots were used to visualize survival differences based on the determined cutoff values.

### 2.2. Differential Expression Analysis

All data processing steps and statistical analyses were conducted using R v3.5.2 (http://www.r-project.org, accessed on 1 August 2024). RNA sequencing (RNA-seq) data were obtained from the Cancer Genome Atlas (TCGA, https://cancergenome.nih.gov/, accessed on 1 August 2024). For expression analyses, RNA-seq HTSeq count data generated by the Illumina HiSeq 2000 RNA Sequencing Version 2 platform were utilized. The raw count data were normalized using the “DESeq” package, which is based on the negative binomial distribution. The Bioconductor “AnnotationDbi” package (http://bioconductor.org/packages/AnnotationDbi/, accessed on 1 August 2024) was used to annotate Ensembl transcript IDs with gene symbols. A second scaling normalization was performed to set the mean expression of all genes in each patient sample to 1000, thereby reducing batch effects. Expression between normal and tumor samples was compared using a Mann–Whitney test.

Methylation Analysis: Beta values of the methylation status of the *DAPK1* promoter, RNA-Seq data, and clinical data from ovarian serous adenocarcinoma (OV) patients in The Cancer Genome Atlas (TCGA) were downloaded from the TCGA data portal (https://portal.gdc.cancer.gov, accessed on 1 August 2024) on 31 October 2023. Correlations between genomic and survival data were performed as previously described [13]. Methylation status was stratified based on quartiles (1st quartile = low methylation, 2nd and 3rd quartiles = intermediate methylation, 4th quartile = high methylation). To compute overall survival, the patient’s date of death from any cause or the last date the patient was known to be alive was considered. IBM SPSS Statistics v29 (IBM, Ehningen, Germany) was used to calculate the Kaplan–Meier survival curve and log-rank test.

### 2.3. Generation of mRNA

The plasmid template containing the truncated Flag-DAPK1 open reading frame was PCR-amplified using a reverse primer engineered to incorporate a 120-nucleotide poly(A) tail and restore T7 promoter functionality. In vitro transcription was performed using CleanCap^®^ AG (Tebu-Bio) for 5′ capping (Cap 1 structure), with complete substitution of uridine by pseudouridine-5′-triphosphate. Post-IVT, template DNA was degraded using DNase I (Tebu-Bio), followed by purification through silica membrane spin columns (Tebu-Bio) to remove reaction byproducts, including residual template DNA, excess NTPs, and capping reagents. Additional purification steps included Antarctic phosphatase (Tebu-Bio) treatment to eliminate potentially immunogenic 5′-triphosphate groups from uncapped transcripts, followed by phosphatase removal via RNeasy column purification (Qiagen). Final mRNA products were obtained from TriLink BioTechnologies (distributed by Tebu-Bio).

### 2.4. Cell Lines, Primary Cells, and Transfection

Purchased from the DCTD Tumor Repository (Bethesda, MD, USA), the human ovarian cancer cell lines SKOV3, OVCAR-3, OVCAR-4, OVCAR-5, and OVCAR-8 were kept in RPMI 1640 medium (#71870036, Thermo Fisher Scientific, Dreieich, Hessen, Germany), which contained 1% penicillin/streptomycin and 10% fetal bovine serum (FBS; #A5256701, Thermo Fisher Scientific, Dreieich, Germany). Every cell line was cultivated at 37 °C in a humidified incubator with 5% CO_2_.

Primary cells were extracted from OC tissues obtained during in-house surgery at the Department of Gynecology, Medical School, Goethe University, which was approved by the medical ethics committee of the University Hospital Frankfurt am Main in accordance with all relevant ethical regulations (permission number: SGO-1-2017) [14]. Patients signed informed consent forms approved by the responsible authority.

DMEM (Fisher Scientific, Dreieich, Hessen, Germany) supplemented with 1% penicillin/streptomycin (Thermo Fisher Scientific, Dreieich, Hessen, Germany) was used to store the samples. The tissue samples were put on a sterile Petri plate under a laminar flow and carefully rinsed with cold PBS (Thermo Fisher Scientific, Dreieich, Hessen, Germany), and blood and necrotic and fatty inclusions were removed. The tissue was divided for further designated use: for 2D primary cells, for organoids, for IHC, or shock-frozen for later use at −80 °C.

The tissue sample for the 2D primary cells was sliced by scalpel into tiny pieces (1–2 mm) and digested with the same volumes of 10× trypsin (Sigma-Aldrich/Merck, Darmstadt, Hessen, Germany) and a collagenase-1 solution (Worthington, Lakewood, NJ, USA (1 mg/mL, filter sterilized)). Volumes (2–10 mL each) and incubation time (45–60 min) to digest in a 50 mL tube at 37 °C, depending on the tissue size and character, were obtained. Digestion was stopped by adding the two-fold volume of fetal bovine serum (FBS) (Gibco/Thermo Fisher Scientific, Dreieich, Hessen, Germany), and the sample was filtered sequentially through cell strainers (100, 70, and 40 µm), thereby washed several times with PBS and collected into a 50 mL tube. The flow-through was then centrifuged for 5 min at 200× *g*. After discarding the supernatant, the cell pellet was plated in a T75 cell culture flask with a primary cell growth medium (RPMI 1640, Fisher Scientific, Dreieich, Hessen, Germany), 2% FBS (Gibco/Thermo Fisher Scientific, Dreieich, Hessen, Germany), 1% penicillin/streptomycin (Thermo Fisher Scientific, Dreieich, Hessen, Germany), 0.01 µg/mL of EGF (Sigma-Aldrich/Merck, Darmstadt, Hessen, Germany), 20 µg/mL of Insulin (Merck, Darmstadt, Hessen, Germany), 0.5 µg/mL of Hydrocortison (Merck, Darmstadt, Hessen, Germany), 25 ng/mL of Choleratoxin (Merck, Darmstadt, Hessen, Germany), and 1 µg/mL of Amphotericin B (Sigma-Aldrich/Merck, Darmstadt, Hessen, Germany)). If the pellet contained considerable amounts of red blood cells, 8–10 min of lysis at 37 °C was conducted with ammonium–chloride–potassium buffer (red lysis buffer, p. 25).

### 2.5. Ascites

In the case of OC-developed ascites (instead of tissue samples), digestion steps were omitted, and (depending on the ascites volume) the sample was either firstly centrifuged to concentrate for 5 min at 200× *g* or, in the case of low volume, directly filtered (100, 70, and 40 µm cell strainers), and the flow-through was spun down again for 5 min at 200× *g*. The cell pellet was seeded in a T75 cell culture flask with a primary cell growth medium (as described before).

### 2.6. Organoids

After the in-house procedure, tissues were collected and immediately placed in an ice-filled storage medium (Dulbecco’s Modified Eagle’s Medium [DMEM; Thermo Fisher Scientific, Dreieich, Hessen, Germany], supplemented with 1% penicillin/streptomycin [Thermo Fisher Scientific, Dreieich, Hessen, Germany]). Thermo Fisher Scientific, Dreieich, Hessen, Germany, supplied 500 mL of DMEM, 1% penicillin/streptomycin, and 2 g of bovine serum albumin (BSA) as the wash medium, which was used three times to rinse fresh tissues that had been chopped into tiny pieces (2–4 mm). The sample was then allowed to rest on ice for two minutes, after which the supernatant was extracted. Lastly, depending on the size of the sample, the tissue was stored for up to 30–40 min at 37 °C in 5–20 mL of digestion media (20 mL of Advanced DMEM (Thermo Fisher Scientific, Dreieich, Hessen, Germany), 20 mg of collagenase-1 (Worthington, Lakewood, NJ, USA), 5 µM of Y-27632 (Biozol, Eching, Bavaria, Germany)).

The sample was shaken every five to ten minutes, and the state of digestion was monitored under the microscope. As soon as the tissue was broken down into individual cells or tiny clusters of cells, the digestion process was stopped with an equivalent volume of wash media. Next, a 100 μm filter was used to filter the cell suspension, which was then spun down at 200× *g* at 4 °C for 5 min to stop digestion and remove collagenase. In the case of OC-derived ascites, digestion was redundant; the sample was just passed through a 100 µm cell strainer, spun down at 200× *g* at 4 °C for 5 min, and the procedure could continue as follows. After discarding the supernatant, if the pellet appeared red due to remaining erythrocytes, the red blood cells were lysed by ammonium–chloride–potassium buffer (red lysis buffer: 0.15 M NH_4_Cl [Sigma-Aldrich/Merck, Darmstadt, Hessen, Germany], 10 mM KHCO_3_ [Sigma-Aldrich/Merck, Darmstadt, Hessen, Germany] and 0.1 mM ethylenediamine–tetraacetic acid [Carl Roth, Karlsruhe, Baden-Würtemberg, Germany]). Depending on the pellet size, 0.5 to 1 mL of red lysis buffer was added to the cells, which were then allowed to incubate for two to three minutes at room temperature before being washed again two to three times with wash medium to rinse the lysis buffer.

After discarding the supernatant, the pellet was resuspended in a cold wash medium and combined with a triple volume of Base Membrane Extract (Bio-Techne, Wiesbaden, Hessen, Germany) or Matrigel Matrix Basement Membrane (Corning/Thermo Fisher Scientific, Dreieich, Hessen, Germany). The matrix mixture was kept on ice, and 3D droplets (10–20 µL) were quickly set in a 12-well suspension plate. Otherwise, the matrix would start to gel above 10 °C. After turning the plate upside-down, it was incubated for 20–30 min at 37 °C and 5% CO_2_ in a cell culture incubator. When the matrix domes solidified, 1 mL of growth medium (46.7 mL of Advanced DMEM/F12 [Thermo Fisher Scientific, Dreieich, Hessen, Germany], 1% glutamin [Merck, Darmstadt, Hessen, Germany], 1% penicillin/streptomycin [Thermo Fisher Scientific, Dreieich, Hessen, Germany], 10 µM of Y-27632 [Biozol, Eching, Bavaria, Germany], 0.25 µM of A83-01 [Bio-Techne, Wiesbaden, Hessen, Germany], 1× B27 supplement [Thermo Fisher Scientific, Dreieich, Hessen, Germany], 5 mM nicotinamide [Merck, Darmstadt, Hessen, Germany], 1.25 mM N-acetylcysteine [Merck, Darmstadt, Hessen, Germany], 1× N2 supplement [Thermo Fisher Scientific, Dreieich, Hessen, Germany], 1 µM of SB-203580 (p38i) [AdipoGen, Fuellinsdorf, Switzerland], 50 ng/mL of hEGF [Peprotech/Thermo Fisher Scientific, Dreieich, Hessen, Germany], 50 ng/mL of Neuregulin 1 (NRG 1) [Peprotech/Thermo Fisher Scientific, Dreieich, Hessen, Germany], 10 ng/mL of HGF [Peprotech/Thermo Fisher Scientific, Dreieich, Hessen, Germany], 20 ng/mL of IGF [Peprotech/Thermo Fisher Scientific, Dreieich, Hessen, Germany], 10 nM of β-Estradiol [Sigma-Aldrich/Merck, Darmstadt, Hessen, Germany], 200 ng/mL of Primocin [InvivoGen, San Diego, CA, USA], 50 ng/mL of R-Spondin [Peprotech/Thermo Fisher Scientific, Dreieich, Hessen, Germany], and 100 ng/mL of Noggin [Bio-Techne, Wiesbaden, Hessen, Germany] was added to each well and changed twice a week. Passaging depended on the size and density of the organoids.

### 2.7. Transfection

For DNA transfections, we employed either Lipofectamine LTX (Invitrogen, Waltham, MA, USA) or jetPEI transfection reagent (Polyplus, Illkirch, Bas-Rhin, France), according to standard protocols. mRNA transfections were performed using Lipofectamine Messenger MAX (Invitrogen) at a ratio of 1 µg of IVT mRNA to 2 µL of transfection reagent, following the manufacturer’s specifications. Following transfection, ovarian cancer cells were harvested and lysed in RIPA buffer (Thermo Fisher Scientific) for subsequent protein analysis. Cellular lysates were clarified by centrifugation at 15,000× *g* for 5 min at 4 °C to obtain the cytoplasmic protein fraction in the supernatant.

### 2.8. Colony Formation Assay

Cells (2000/well) were seeded in 6-well plates and transfected as described. Following transfection, cells were washed, replenished with fresh medium, and cultured for 14 days to allow colony formation. Colonies were fixed with 70% ethanol and stained with 0.5% Coomassie Brilliant Blue (Bio-Rad, Feldkirchen, Bavaria, Germany). Quantitative analysis was performed by counting visible colonies (>50 cells) using an AxioObserver Z1 microscope (Zeiss, Göttingen, Germany), with documentation achieved via a ChemiDoc MP imaging system (Bio-Rad).

### 2.9. Western Blot (WB) and Antibodies

Protease inhibitor-supplied RIPA buffer (Sigma) was used to lyse cell protein extracts (complete protease inhibitor cocktail, Roche, Mannheim, Rheinland Pfalz, Germany). Using a TransBlot Turbo Transfer System (Bio-Rad, Feldkirchen, Bavaria, Germany), protein extracts (25 μg) were separated using sodium dodecyl sulfate–polyacrylamide gel electrophoresis and then transferred onto polyvinylidene difluoride membranes. Tris-buffered saline with 0.05% Tween^®^ 20 (Sigma, Frankfurt, Hessen, Germany) and 2% BSA (Thermo Fisher Scientific, Dreieich, Hessen, Germany) was used to block the membranes.

Protein concentrations were determined using absorption-based quantification (ROtiquant, Carl Roth GmbH, Karlsruhe, Germany) according to the manufacturer’s protocol. Measurements were performed using a PerkinElmer Victor X4 plate reader. For standardization, equal protein loading was verified by β-actin expression, which served as the loading control for normalizing protein expression levels across samples.

Antibodies were used at the following concentrations: DAPK1 (ab109382) (1:1000; Abcam, Cambridge, United Kingdom), mouse monoclonal PLK1 (F-8:sc-17783) (1:1000; Santa Cruz, Biotechnology Heidelberg, Germany), p53 (sc-126) (1:1000; Santa Cruz), MDM2 (sc-965) (1:1000; Santa Cruz), β-Actin (AC-15:A1978) (1:200.000; Sigma-Aldrich, Taufkirchen, Germany), Flag (M2:A8592) (1:1000; Sigma-Aldrich), p16 (#18769) (1:1000; Cell Signaling, Danvers, MA, USA), Noxa (#14766) (1:1000; Cell Signaling), p14ARF (#74560) (1:1000; Cell Signaling), Caspase-2 (#2224) (1:1000; Cell Signaling, Danvers, MA, USA), LC3A/B (#12741) (1:1000; Cell Signaling), p53-pS20 (#9287) (1:1000; Cell Signaling, Danvers, MA, USA), p21 (#2947) (1:1000; Cell Signaling), PARP (#9542) (1:1000; Cell Signaling, Danvers, MA, USA), Puma (#98672) (1:1000; Cell Signaling, Danvers, MA, USA), Bax (AF820) (1:1000; R&D systems, MN, USA), and HRP-conjugated secondary antibodies (1:5000; GE Healthcare and Jackson Laboratory, Bar Harbor, ME, USA). The ECL WB Substrate (Millipore, Burlington, MA, USA) was used for detection.

### 2.10. Cell Viability and Proliferation Assays

The Caspase-Glo 3/7 assay kit (Promega, Waldorf, Hessen, Germany) was used in accordance with the manufacturer’s guidelines. The mean value ± standard deviation (*n* = 3) is displayed for the measured luminescence (RLU). Non-synchronized cells were exposed to the test chemicals for the specified durations in order to perform the apoptotic experiments. As directed by the manufacturer, fluorescence was used as a readout for cell viability experiments using the Cell Titer-Blue Cell Viability Assay (Promega) at excitation and emission wavelengths of 562 nm and 615 nm. The significance of the variations among data populations was evaluated using Student’s two-tailed test (* *p* ≤ 0.05; ** *p* ≤ 0.01; *** *p* ≤ 0.001). The cell proliferation experiments were carried out with the Cell Titer-Blue Cell Viability Assay (Promega). To put it briefly, DAPK1 mRNA or control mRNA was transfected into cells that had been seeded on 96-well plates. Afterward, a Victor X4 Multilabel Counter (Victor X4, PerkinElmer, Rodgau, Hessen, Germany) was used to measure the fluorescence at the designated time points.

### 2.11. Immunofluorescence Assay

Cells were transfected with the Flag-DAPK1 vector and DAPK1-mRNA and seeded on coverslips. Twenty-four hours later, cells were washed for 5 min with PBS and fixed with 4% paraformaldehyde/0.5% Triton-X-100 for 20 min at room temperature. The following primary antibodies were used for staining: Flag, alpha-tubulin, and LC3A/B. Alexa 555, Alexa 488, and Cy3 secondary antibodies were obtained from Abcam, Inc. DNA was stained with DAPI (Roche Diagnostics). Images were captured using an AxioObserver Z1 microscope with an HCX PL APO CS 63.0 × 1.4 oil UV objective (Carl Zeiss AG) was used.

### 2.12. Statistical Methods

All experiments were performed at least in triplicate. Standardization and statistical analyses were performed as previously described [15]. Statistical analyses were performed using Microsoft Excel and GraphPad Prism v 7.05. For paired *t*-tests, all experimental groups were compared with their respective groups. Student’s *t*-test and the Wilcoxon test were used to determine statistical significance between the two groups unless otherwise indicated. A *p* value < 0.05 was considered statistically different. Significant differences (* *p* ≤ 0.05, ** *p* ≤ 0.01, and *** *p* ≤ 0.001) are indicated by asterisks.

## 3. Results

### 3.1. Screening for Novel Tumor Suppressor Genes in Ovarian Cancer

**Figure 1:**
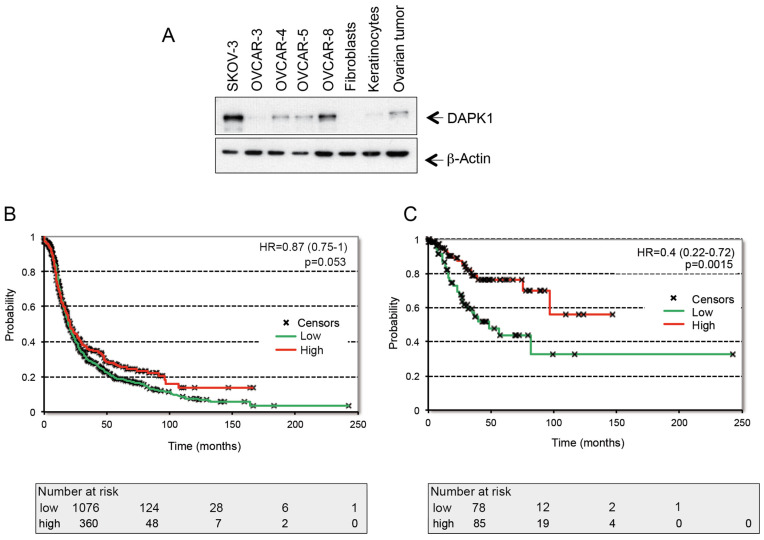
DAPK1 expression in high-grade serous ovarian cancer (HGSOC) cells and its prognostic role. (**A**) Expression analysis of DAPK1 in ovarian cancer cell lines and primary cells by Western Blot (WB). Cell lysates were immunoblotted for DAPK1 and β-Actin. For all panels, one image representative of three independent experiments is shown. (**B**) Patients were divided into groups with low and high levels of DAPK mRNA expression based on the best cutoff option. Using the Kaplan–Meier plotter database, survival curves for progression-free survival for (**B**) stages I–IV and (**C**) early stage I + II tumors of patients with OC treated with debulking surgery and CT are shown. Every graphic has log-rank *p*-values, the hazard ratio (HR), and 95% confidence intervals. (The uncropped blots are shown in Figure S4/Figure 1A).

Although new drug candidates [16] and changes to the scheduling and administration of chemotherapy have improved outcomes in ovarian cancer patients to a certain degree, a therapeutic ceiling is being reached with these approaches, leading to a therapeutic situation where, during the last 20 years, only marginal improvement in 5-year overall survival has been achieved. Drug resistance is one of the most severe problems in clinical oncology, in particular, in the case of ovarian cancer, yet the classical strategy to identify anticancer drugs usually encompasses screening procedures that predominantly consider small-molecule inhibitors. To broaden the spectrum and quality of new anti-ovarian cancer agents, we screened for the targeted reactivation of putative tumor suppressor genes and the exploration of their role in HGSOC using vector-prone approaches and IVT-mRNAs. To this end, we screened the expression patterns of different putative candidate genes, including *DAPK1*, in gynecological cancer cell lines and primary cancer cells. Our previous investigation evaluated DAPK1 expression in several gynecological cancer cell lines (cervical, ovarian, and breast) and the cell lines of different origin (colon, lung, and leukemia), as well as primary normal cells (keratinocytes and fibroblasts) [17]. While the analysis of the cervical HeLa cell line and the lung cancer cell line A459 provided evidence for highly frequent DAPK1 expression [17], breast cancer cell lines showed very low levels of the DAPK1 protein. In this study, we could corroborate strong DAPK1 expression in the ovarian cancer cell line SKOV-3 (low-grade serous ovarian cancer) (Figure 1A). In contrast, the DAPK1 signal in HGSOC cells, such as OVCAR-3, was below the detection limit or low in OVCAR-4, -5, and -8 cells (Figure 1A).

To explore the significance of DAPK1 expression in ovarian cancer patients, we used a database including 1436 patient samples with available progression-free survival (PFS) times to evaluate the prognostic potential of *DAPK1*. The probe set 203139_at was used to determine the expression of *DAPK1*. The aggregate characteristics of patients (stages I–IV) and for patients (stages I and II) are provided in Table 1. When analyzing survival in all patients (stages I–IV), a borderline significance was observed (HR = 0.87, *p* = 0.053; median survival in the low-expression cohort = 19.4 months, median survival in the high-expression cohort = 21 months) (Figure 1B). When restricting to patients only in stages I and II (*n* = 163), there was a significant correlation with survival (HR = 0.4, *p* = 0.0015, median survival in the low-expression cohort = 18.3 months, median survival in the high-expression cohort = 75.6 months) (Figure 1C), suggesting *DAPK1* expression is a prognostic marker in the early stages of ovarian cancer.

### 3.2. The Regulation of DAPK1 Expression and Its Role in the Survival of HGSOC Patients

**Figure 2:**
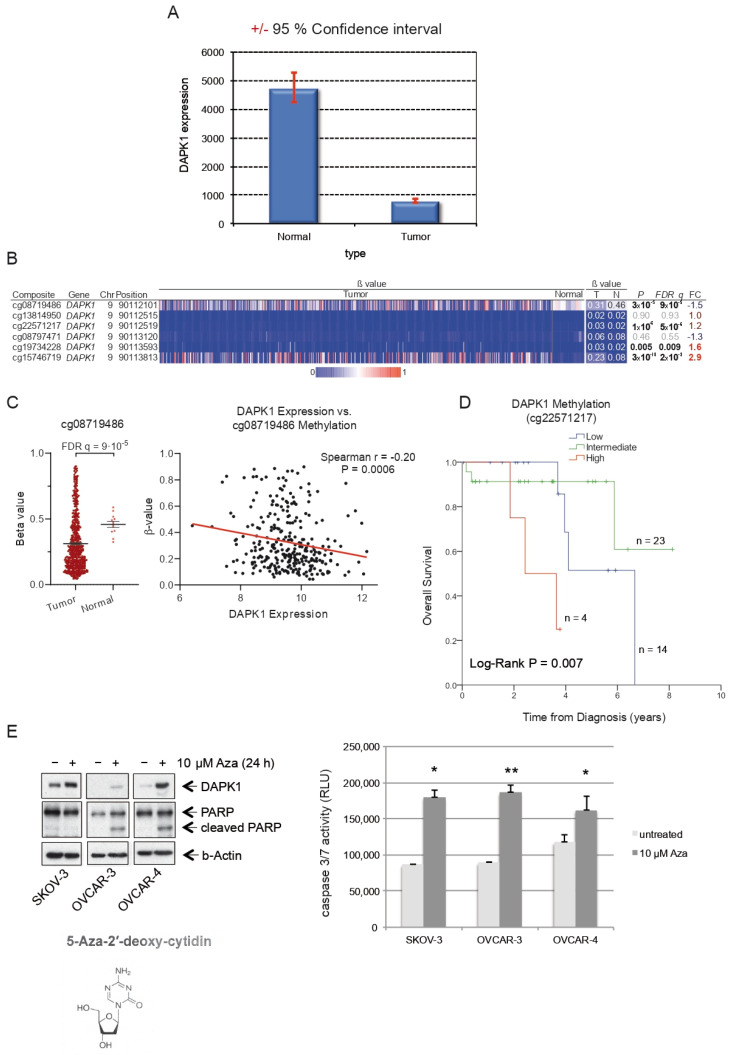
Epigenetic regulation of DAPK1 expression. (**A**) RNAseq-based expression of DAPK1 shows lower expression in ovarian cancer patients (*n* = 374) compared to normal tissues (*n* = 133). Mann–Whitney U-test *p* < 1 × 10^−16^. (**B**) Methylation status at the DAPK1 promoter for 600 tumors compared to 12 normal samples from OV-TCGA. FC, fold change. (**C**) Comparison of the Beta values for the tumor and normal samples and their inverse correlation to DAPK1 gene expression. (**D**) Kaplan–Meier survival curves of OV-TCGA patients with low-stage (I and II) disease, showing high methylation (4th quartile, red) in the DAPK1 promoter at cg22571217, are significantly associated with poorer overall survival. The first quartile is depicted in blue, whereas the second and third quartiles are shown combined in green. (**E**) Ovarian cancer cell lines were treated with 10 µM of 5-Azacytidine for 24 h. Cell lysates were immunoblotted for DAPK1, PARP, and β-Actin (upper left panel). The chemical structure of 5-Azacytidine (Aza) is shown (lower left panel). A Caspase-Glo 3/7 assay was used to measure the Aza-dependent Caspase-3/7 activity (right panel). *p* < 0.05 for *; *p* < 0.01 for **. Student’s two-tailed, unpaired *t*-test. (The uncropped blots are shown in Figure S4/Figure 2C).

In a large cohort of 507 ovarian tissues, comparing DAPK1 expression in benign and malignant epithelial ovarian tissues revealed a significant downregulation of *DAPK1* in cancer tissues by a factor of 6.1 (Figure 2A). To further investigate the regulation of *DAPK1* expression, we explored its promoter region for potential regulatory mechanisms, including epigenetic alterations. The hypermethylation of CpG sites was considered as a procedure to trigger *DAPK1* expression (Figure 2B). Several markers showed significantly higher Beta values in tumors than in normal samples (FDR *q* = 5 × 10^−6^ for cg22571217, FDR *q* = 0.009 for cg19734228, and FDR *q* = 2 × 10^−9^ for cg15746719). As expected, there was a significant negative correlation between *DAPK1* promoter methylation and *DAPK1* expression by RNA-Seq (Figure 2C). In addition, ovarian cancer TCGA patients with low stages (I and II) and a high methylation of *DAPK1* at the cg22571217 site displayed poor overall survival (log-rank *p* = 0.007) (Figure 2D), supporting the role of the epigenetic regulation of *DAPK1* in ovarian cancer patients.

In our subsequent evaluation, which aimed to identify the mechanistic background of low DAPK expression in ovarian cancer cells, we used 5-Azacytidine, an antineoplastic nucleoside analog that functions as an epigenetic modifier by inhibiting DNA methyltransferase, to treat ovarian cancer cells. By this means, we could induce a remarkable increase in DAPK1 expression in all three ovarian cell lines (Figure 2E, left panel), associated with an increase in apoptotic activity (Figure 2E, right panel), suggesting that DAPK1 transcription and cell fate are controlled by epigenetic modifications.

### 3.3. Exogenous Expression of Wild-Type DAPK1 Induces Cell Death in HGSOC Cell Lines

**Figure 3:**
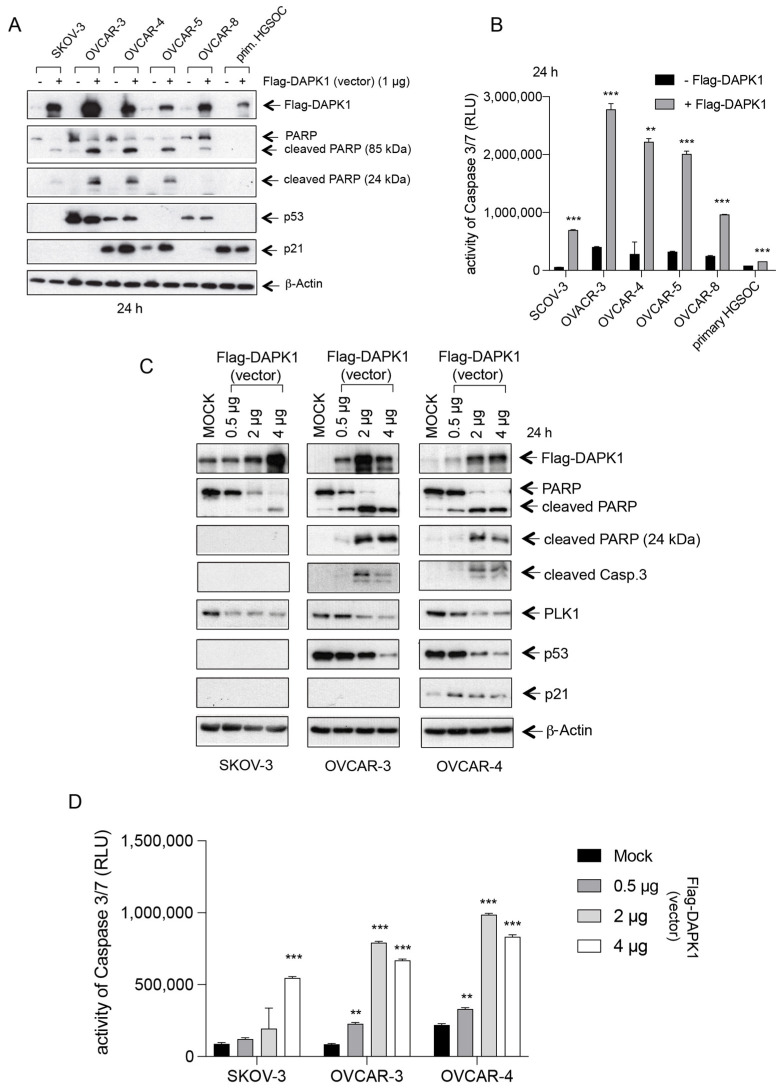
Mammalian vector-based expression of full-length DAPK1 in HGSOC cells induces apoptosis. (**A**) A mammalian vector was used to express full-length DAPK1 in different ovarian cancer cell lines and primary HGSOC cells. Using Flag, PARP, p53, p21, and β-Actin antibodies, cell lysates were submitted to WB. (**B**) A Caspase-Glo 3/7 assay was used to measure the Caspase-3/7 activity of cell lines and primary cells transfected with recombinant DAPK1-expressing vectors, as depicted in (**A**). *p* < 0.01 for ** and *p* < 0.001 for ***. Student’s two-tailed, unpaired *t*-test. (**C**) Increasing doses of recombinant DAPK1-expressing vectors were used to transfect ovarian cancer cell lines. Cell lysates were submitted to WB using Flag, PARP, Caspase 3, PLK1, p53, p21, p14ARF, pMLC-2, and β-Actin antibodies. (**D**) A Caspase-Glo 3/7 assay was used to measure the dose-dependent Caspase-3/7 activity of cells depicted in (**C**). *p* < 0.01 for ** and *p* < 0.001 for ***. Student’s two-tailed, unpaired *t*-test. (The uncropped blots are shown in Figures S5–S7).

Considering the upregulated promoter methylation of DAPK1 and its prognostic power in the early stages of the disease, we wondered whether DAPK1 reactivation might have anti-proliferative potential in ovarian cancer. To this end, we evaluated the effects of the re-expression of wild-type (wt), full-length, Flag-tagged DAPK1 on cell viability using high-grade and non-high-grade ovarian cancer cell lines. Reduced cell viability, as measured by PARP cleavage in cell lines and increased Caspase 3/7 activity in cell lines and primary tumor cells, was observed upon the mammalian vector-based expression of DAPK1 (Figure 3A,B). The induction of apoptosis, as indicated by the cleavage of PARP and Caspase-3, was concentration-dependent, whereas a control vector was ineffective (Figure 3C,D). The expression of PLK1, a key regulator of cell cycle progression and a marker of cellular proliferation [18,19,20,21,22,23], was inversely correlated with the vector-prone increase in DAPK1 re-expression (Figure 3C), supporting an anti-proliferative role of DAPK1 in ovarian cancer. Cell lines expressing the classical p53 target gene, p21 [24,25], exhibited increased p21 levels upon DAPK1 expression compared to mock-transfected cells (Figure 3A,C). With rising levels of apoptosis, the level of p21 began to decrease (Figure 3C). The vector-based rescue of DAPK1 expression in the HGSOC cells, OVCAR-3 and OVCAR-8, induced the upregulation of p14ARF expression, consistent with the role of DAPK1 in promoting the expression of p14ARF [11] (Figure 3C). In addition, we observed increased myosin regulatory light chain-2 (MLC-2) phosphorylation, a direct target of DAPK1, indicating that the vector-based restoration of DAPK1 retains full activity (Figure 3C).

The amount of transfected Flag-tagged DAPK-expressing vector correlated with the level of Caspase 3/7 activity, supporting a dose-dependent apoptotic response (Figure 3D). The reduced levels of Caspase 3/7 activity in cells transfected with 4 μg of Flag-DAPK1 compared to cells transfected with 2 μg of Flag-DAPK1 are likely due to the rising levels of cell death associated with overall protein decay (Figure 3D). Interestingly, in SKOV3 cells, which lack p53 expression, we observed an important increase in cell death following DAPK1 reactivation, as evidenced by PARP cleavage (Figure 3C, left panel) and a significant induction of Caspase 3/7 activity (Figure 3D). These findings strongly suggest a p53-dependent and -independent role of DAPK1 in promoting apoptosis in ovarian cancer cells.

**Figure 4:**
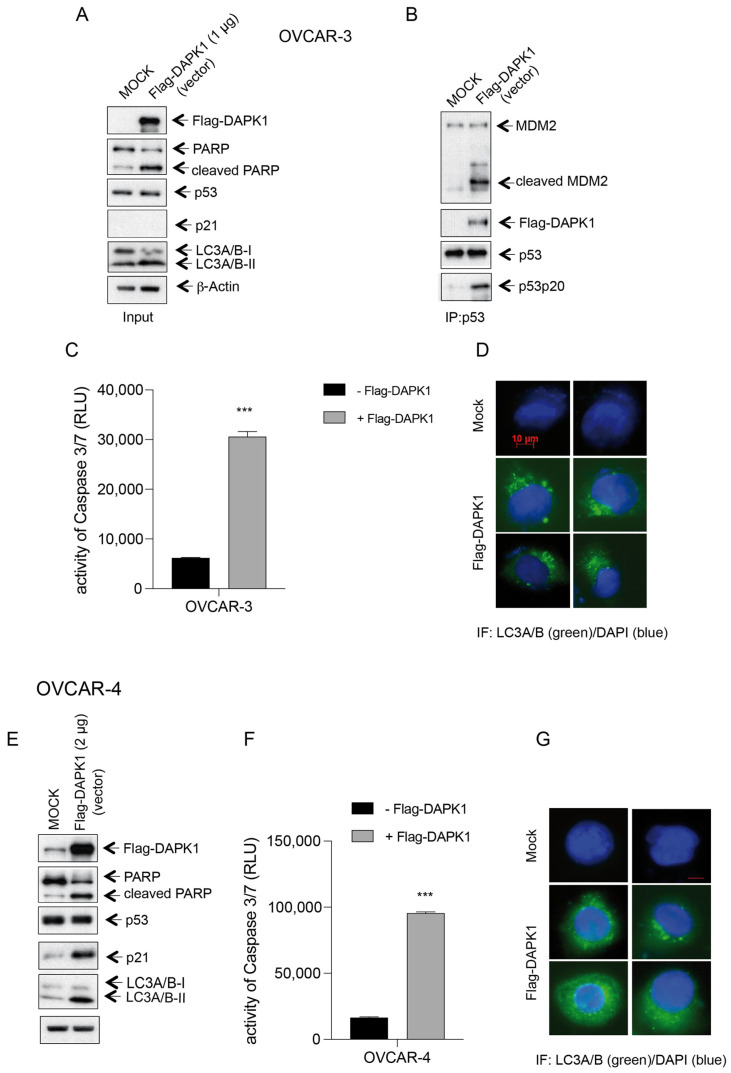
DAPK1 expression induces MDM2 cleavage in OVCAR-3 cells and phosphorylates p53 at Ser-20. (**A**) Lysates of cells transfected with 1 μg of a full-length DAPK1-expressing vector were subjected to SDS-PAGE and stained using antibodies for Flag, PARP, p53, p21, LC3A/B-I, LC3A/B-II, and β-Actin. (**B**) The lysates were subjected to IP using p53-specific antibodies, followed by Western blotting with antibodies for MDM2, Flag, p53, and p53pS20. (**C**) A Caspase-Glo 3/7 assay was used to measure the Caspase-3/7 activity of OVCAR-3 cells transfected with a recombinant DAPK1-expressing vector shown in (**A**). *p* < 0.001 for ***. Student’s two-tailed, unpaired *t*-test. (**D)** Representative immunofluorescence images showing the morphology of LC3-positive structures after the expression of exogenous Flag-DAPK in the HGSOC OVCAR-3 cell line (green: LC3-positive structures, blue: DNA stained with DAPI). Scale bar = 10 µm. (**E**) DAPK1 expression increases p21 levels and apoptosis in OVCAR-4 cells. Lysates of the cells transfected with 2 μg of a full-length DAPK1-expressing vector were subjected to an SDS-PAGE and stained using antibodies for Flag, PARP, p53, p21, LC3A/B-I, LC3A/B-II, and β-Actin. The exogenous expression of Flag-DAPK1 led to an increase in the cytoplasmic LC3-II levels, suggesting enhanced autophagy. (**F**) A Caspase-Glo 3/7 assay was used to measure the Caspase-3/7 activity of OVCAR-4 cells transfected with a recombinant DAPK1-expressing vector. *p* < 0.001 for ***. Student’s two-tailed, unpaired *t*-test. (**G**) Representative immunofluorescence images showing the morphology of LC3-positive structures after the expression of exogenous Flag-DAPK in the HGSOC OVCAR-4 cell line (green: LC3-positive structures, blue: DNA stained with DAPI). Scale bar = 10 µm. (The uncropped blots are shown in Figure S8).

The tumor suppressor gene p53, which is mutated in 50% of human cancers and more than 95% of HGSOCs, is a transcriptional regulator and controls necrotic and apoptotic pathways in human cells via transcription-dependent and transcription-independent mechanisms [26,27]. Human p53-Serine-20 (pS20) is a target of DAPK1 [28]. It was reported that DAPK1 might bind to p53 to mediate apoptosis in neuronal cells [29]. To explore a direct putative regulation of p53 by DAPK1 in ovarian cancer cells, we performed an immunoprecipitation (IP) of p53 from cells overexpressing Flag-DAPK1 (Figure 4A,B) and identified DAPK1 as an interacting partner (Figure 4B). Remarkably, only in Flag-DAPK1-expressing cells, the phosphorylation of p53 at Ser-20, a target of DAPK1, could be demonstrated (Figure 4B), suggesting p53 as a substrate of DAPK1 in ovarian cancer cells. In addition, we analyzed the mouse minute 2 protein (MDM2), a critical regulator of the tumor suppressor protein p53 [30,31]. MDM2 is a negative regulator of p53, binding to it and promoting its degradation via the ubiquitin–proteasome pathway. The IP experiment revealed MDM2 to be bound to p53 in mock-transfected and Flag-DAPK1-expressing OVCAR-3 cells (Figure 4B). Importantly, only in DAPK-expressing OVCAR-3 cells were uncleaved MDM2 and high amounts of cleaved MDM2 found to be associated with p53, supporting the model that the p53 binding to cleaved, inactive MDM2 could support the stabilization of p53 (Figure 4B). The stabilization of p53 correlated with enhanced apoptotic activity in DAPK1-expressing OVCAR-3 cells, confirming the reactivation of apoptotic signaling pathways (Figure 4C).

LC3 is a key protein in autophagosome formation and maturation [32]. LC3 exists in two forms: LC3-I (the cytosolic form) and LC3-II (the lipidated form that associates with autophagosome membranes) [32]. It has been shown that the kinase-dependent phosphorylation of LC3 can influence the conversion of the LC3-I form to its lipidated, autophagosome-associated LC3-II form, which is essential for the elongation and closure of autophagosomes, thus regulating the autophagy process [33]. We found that the expression of exogenous DAPK1 in OVCAR-3 cells increased the autophagosome-associated LC3 A/B-II form (Figure 4A) and increased the LC3 puncta in the cytoplasm of the transfected cells (Figure 4D).

Similar experiments in additional HGSOC cell lines like OVCAR-4 supported growth inhibition (upregulation of p21 expression) and the induction of apoptosis (increased PARP cleavage and increased Caspase 3/7 activity) upon Flag-DAPK1 expression (Figure 4E,F). Again, an increase in the autophagosome-associated LC3 A/B-II form (Figure 4E) and the LC3 puncta in the cytoplasm of the DAPK1-expressing cells was observed (Figure 4G). These data suggest that the reactivation of DAPK1 functions by the re-expression of recombinant full-length DAPK1 reduces cellular proliferation associated with rising cell death and altered autophagy regulation in HGSOC cells.

### 3.4. Design, Generation, and Characterization of Truncated DAPK Clones for Efficient Expression in HGSOC Cells

**Figure 5:**
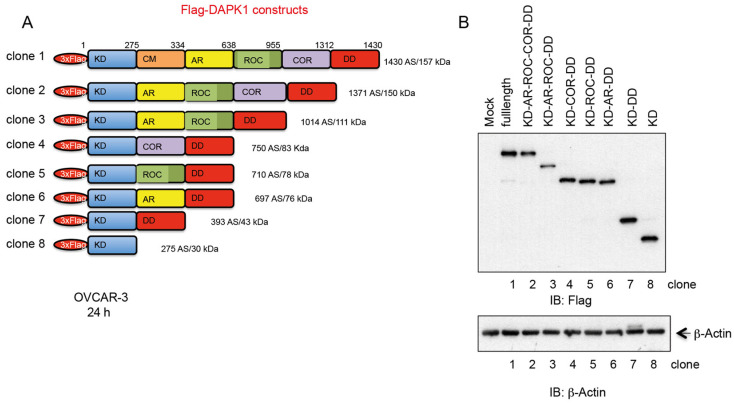
A series of truncated DAPK1 clones was tested for its apoptosis-inducing potential in ovarian cancer cells. (**A**) The DAPK1 functional domains, the kinase domain (KD), the Ca^2+^/CaM autoregulatory domain (CM), the ankyrin repeats (AR), the Ras of complex protein (ROC)—C-terminal of ROC (COR) domain, and the death domain (DD) are shown. (**B**) SDS-PAGE was used to separate full-length DAPK and its deletion clones 2–8, as depicted in (**A**). (The uncropped blots are shown in Figure S9).

In replacement therapy strategies that utilize mammalian vectors to deliver functional proteins for compensating missing or defective gene products, previous studies have shown that reducing the length of recombinant DNA vectors improves transfection efficiency, enhances translational output, and increases the long-term retention of the transgene in target cells [34,35,36]. Considering the relatively long open reading frame of DAPK1, encompassing 4290 base pairs, the aspects of the recombinant vector length are of considerable importance for achieving sufficient and long-lasting DAPK1 expression in cancer cells using mammalian DNA vectors or IVT-mRNA within the context of a rescue strategy. To this end, a series of triple Flag-tagged truncated clones representing different subdomains of DAPK1 (KD, kinase domain; Ca^2+^/CaM, calcium/calmodulin; AR, ankyrin repeats; ROC, Ras of complex protein; COR, C-terminal of ROC; DD, death domain) was generated and expressed in ovarian cancer cells (Figure 5A,B) to evaluate their expression efficiency. Moreover, we determined whether full-length DAPK1 restoration is necessary or if truncated variants are sufficient to sensitize HGSOC cells to Paclitaxel treatment.

### 3.5. Reactivation by Full-Length or by Truncated DAPK1 Sensitizes Ovarian Cancer to Chemotherapeutics

**Figure 6:**
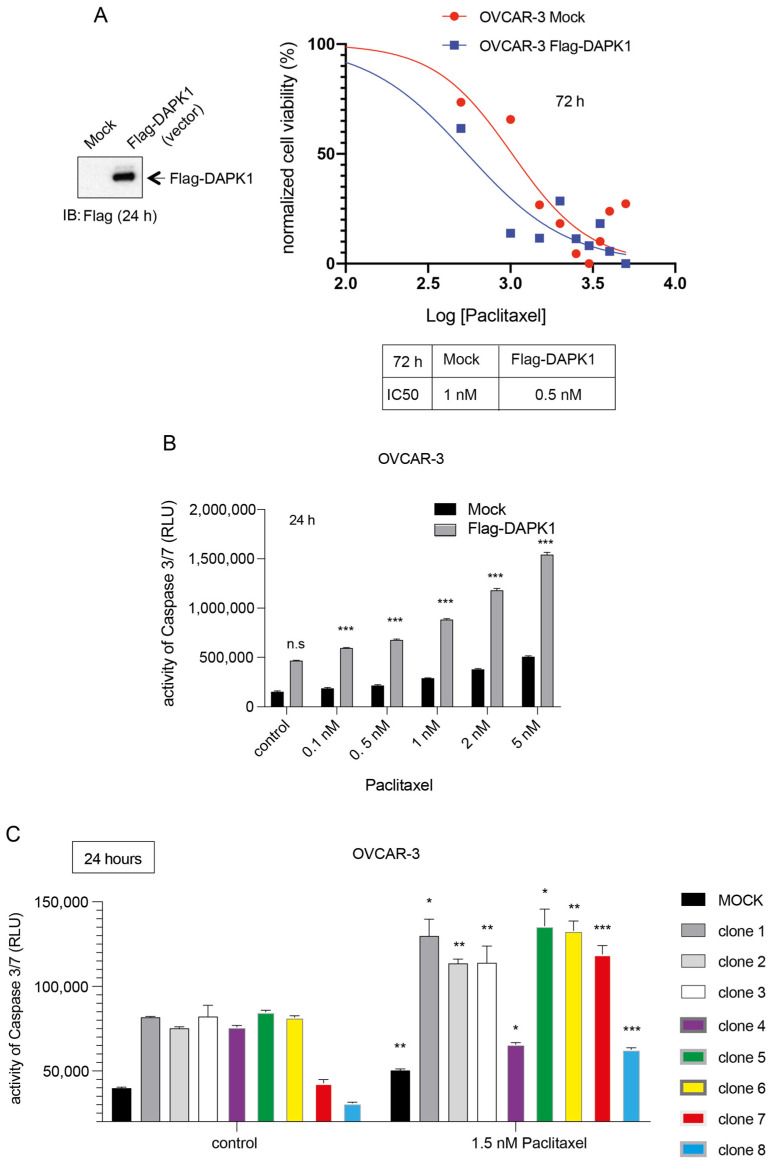
Effects of truncated DAPK1 clone expression on the survival of OVCAR-3 cells. (**A**) Lysates of mock-treated OVCAR-3 cells and OVCAR-3 cells transfected with a full-length DAPK1-expressing vector were subjected to SDS-PAGE and stained using antibodies for Flag-DAPK1. Cells were treated with increasing concentrations of Paclitaxel, and their proliferation was measured using a CellTiter-Blue Cell Viability assay over 72 h, with measurements taken at 72 h. The proliferation rates of the treated cells, an indicator of cell viability, were normalized against their respective DMSO-treated counterparts (vehicle control  =  0 on the *x*-axis) and subsequently used to calculate the IC_50_ values for each cell type. The plots represent the IC_50_ values at the 72 h time point. (**B**) A Caspase-Glo 3/7 assay was used to analyze OVCAR-3 cells expressing a vector-based Flag-tagged full-length DAPK1 fusion protein upon treatment with increasing doses of Paclitaxel. *p* < 0.001 for ***. Student’s two-tailed, unpaired *t*-test. (**C**) A Caspase-Glo 3/7 assay was used to study OVCAR-3 cells (untreated, 1.5 nM Paclitaxel-treated) that were mock-transfected or transfected with DAPK1 subclone (1–8)-expressing vectors. *p* < 0.05 for *, *p* < 0.01 for **, and *p* < 0.001 for ***. Student’s two-tailed, unpaired *t*-test.

The treatment with Cisplatin or Carboplatin combined with Paclitaxel is part of the gold standard for ovarian cancer patients [37]. Therefore, our next objective was to establish the sensitivity of HGSOC cells to standard therapy in wt OVCAR-3 cells that do not express DAPK1 (Figure 1A) and in mammalian vector-transfected OVCAR-3 cells re-expressing DAPK1. To investigate this, we first subjected OVCAR-3 wt and full-length DAPK1-expressing OVCAR-3 cells to escalating concentrations of Paclitaxel and assessed their proliferation over 72 h (Figure 6A,B). We observed that full-length DAPK1 expression sensitized OVCAR-3 cells to Paclitaxel treatment (72 h), reducing the IC_50_ from 1 nM to 0.5 nM while augmenting the apoptotic response compared to wild-type cells (Figure 6A,B). Truncated, short forms of DAPK1, which may allow for improved uptake, translation, and stability in human cells, were also tested for their ability to sensitize ovarian cancer cells to Paclitaxel treatment (Figure 6C). Subjecting mock-transfected OVCAR-3 wt cells and OVCAR-3 cells transfected with full-length DAPK1 or with truncated forms of DAPK1 to 1.5 nM of Paclitaxel showed that the clones 1 (full-length), 2 (KD-AR-ROC-COR-DD), 3 (KD-AR-ROC-DD), 5 (KD-ROC-DD), 6 (KD-AR-DD), and 7 (KD-DD) induced the most prominent apoptotic response compared to transfected and untreated controls (Figure 6C). We could confirm the sensitization to Paclitaxel in OVCAR-4 and OVCAR-8 cells that were both transfected with full-length DAPK1 in comparison to their wt counterparts (IC_50_ wt vs. OVCAR-4 full-length DAPK1 (72 h): 3.25 nM vs. 2.89 of nM Paclitaxel; IC_50_ wt vs. OVCAR-8 full-length DAPK1 (48 h): 2.8 nM vs. 1.4 nM of Paclitaxel) (Supplementary Figure S1A,B). In addition, we studied stably transfected cell lines OVCAR-5 and -8 expressing full-length DAPK1 (Supplementary Figure S2A,B). Treating both cell lines with escalating doses of Paclitaxel supported the significant sensitizing function of DAPK1 expression (Supplementary Figure S2A,B). As shown in Figure 1A, OVCAR5 cells express a very low level of DAPK compared to OVCAR-8. Therefore, we expected that with the exogenous expression of DAPK1, a stronger apoptotic response would be observed in OVCAR-5 than in OVCAR-8, given their medium DAPK1 expression. This expectation could be confirmed in this experiment.

**Figure 7:**
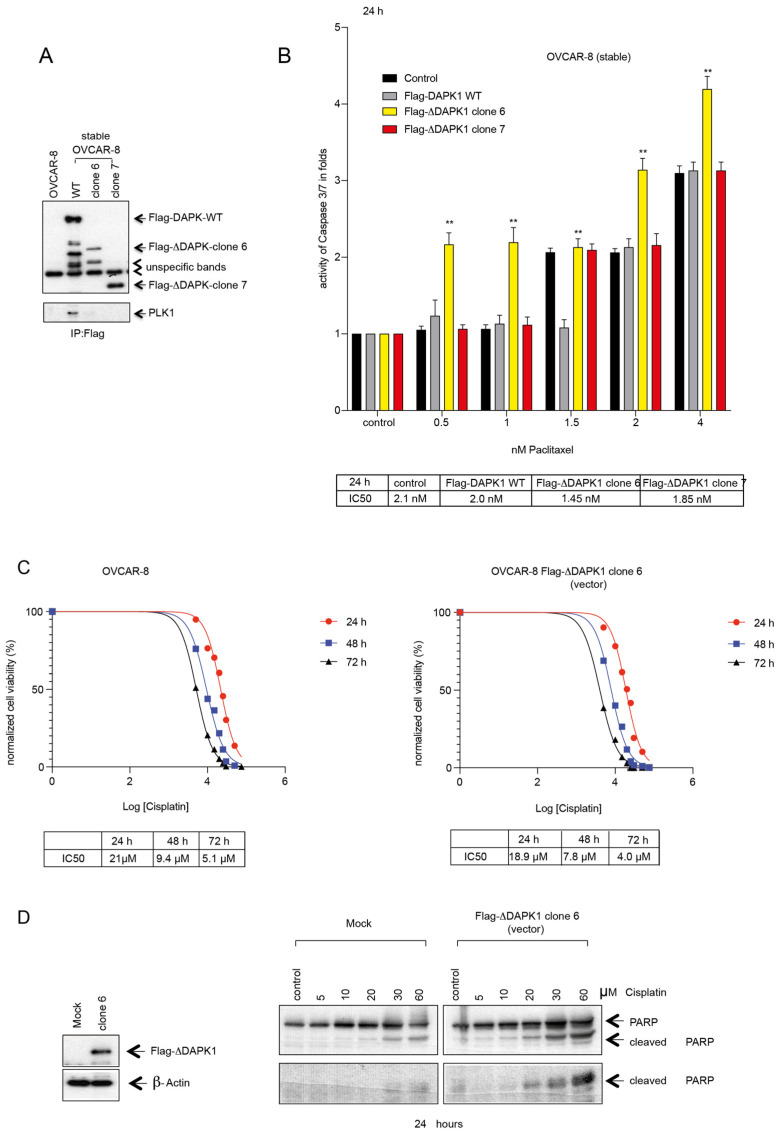
Impact of stably transfected truncated forms of DAPK1 (∆DAPK1 clone 6 and ∆DAPK1 clone 7) on the survival of HGSOC cells under Paclitaxel treatment. (**A**) OVCAR-8 cells stably transfected with a vector expressing full-length DAPK1 or ∆DAPK1 clone 6, or ∆DAPK1 clone 7, were treated with increasing concentrations of Paclitaxel. Lysates of stably expressing cells were subjected to an SDS-PAGE and stained using antibodies for Flag-DAPK1 and PLK1. (**B**) A Caspase-Glo 3/7 assay was used to study OVCAR-8 cells, stably expressing full-length DAPK1 wt or ΔDAPK1/clone 6 or ΔDAPK1/clone 7, treated with increasing doses of Paclitaxel. *p* < 0.01 for **. Student’s two-tailed, unpaired *t*-test. The plots represent the IC_50_ values at 24 h. (**C**) Sensitivity of stably transfected OVCAR-8 cells expressing truncated DAPK1 (∆DAPK1 clone 6) under Cisplatin treatment. OVCAR-8 cells (mock-transfected) and OVCAR-8 cells transfected with ∆DAPK1 (clone 6) were treated with increasing concentrations of Cisplatin, and their proliferation was measured using a Cell Titer-Blue Cell Viability assay throughout 72 h, with measurements taken at 24 h, 48 h, and 72 h. The proliferation rates of the treated cells, an indicator of cell viability, were normalized against their respective DMSO-treated counterparts (vehicle control  =  0 on the *x*-axis) and subsequently used to calculate the IC_50_ values for each cell type. The plots represent the IC_50_ values at different time points. (**D**) Using Flag-DAPK1, PARP, and β-actin antibodies, cell lysates were subjected to Western blot analysis. (The uncropped blots are shown in Figure S10).

Moreover, we tested the sensitizing potential of the truncated versions of DAPK1 (Figure 5A). To this end, OVCAR-8 cells were first stably transfected with wt DAPK1, clone 6, or clone 7 (Figure 7A). While stably expressed Flag-DAPK1 could be co-precipitated with PLK1, confirming our previous data [17], an interaction of Flag-DAPK1 with PLK1 was not observed in clones 6 and 7, suggesting a functional difference between full-length DAPK1 and its truncated relatives (Figure 7A). Subsequently, OVCAR-8 cells stably expressing clones 6 or 7 were treated with increasing doses of Paclitaxel (Figure 7B). While stably integrated clone 6 inhibited cellular proliferation and induced an apoptotic response within 24 h, the apoptotic response to the expression of the integrated clone 7 was generally weaker (Figure 7B). Taken together, by using OVCAR-5 and -8 cells stably transfected with full-length or truncated DAPK1 (Figure 7A,B and Figure S2), we could corroborate our results from the transient transfections of different HGSOC lines like OVCAR-3, -4, and -8 cells (Figure 6, Figure 7A,B, Figure S1 and Figure S2), demonstrating that the reactivation of full-length DAPK1 or the expression of certain truncated forms of DAPK1, such as clone 6, is suited to increase the sensitivity of HGSOC cells to Paclitaxel treatment.

Due to the relevance of Carboplatin or Cisplatin combined with Paclitaxel as part of the standard chemotherapy employed to treat ovarian cancer patients, we also determined the sensitivity of OVCAR-8 cells expressing truncated DAPK1 to Cisplatin. We observed that OVCAR-8 cells expressing clone 6 displayed increased sensitivity to Cisplatin compared to their mock-transfected counterparts (IC_50_ mock-transfected vs. clone-transfected OVCAR-8 cells (72 h): 5.1 μM vs. 4.0 μM) (Figure 7C). The examination of apoptotic activity in DAPK1-clone 6-expressing cells supported the sensitization to Cisplatin, as evidenced by elevated levels of PARP cleavage compared to mock-treated cells (Figure 7D). Collectively, the results suggest that the functional reactivation of DAPK1 or partial functional reactivation by the vector-based expression of wt DAPK1 or DAPK1/clone 6 effectively induces death in HGSOC cells in a dose-dependent manner, supporting sensitization to Paclitaxel and Cisplatin treatment and effectively overcoming their resistance.

### 3.6. IVT ΔDAPK1-mRNA-Based Activation of p53 and Sensitization of Ovarian Cancer Cells to Standard Therapeutics

In vitro-transcribed mRNA (IVT-mRNA) is emerging as a promising tool in cancer therapy. IVT-mRNA offers advantages like rapid production, customizable sequences, and a favorable safety profile compared to DNA-based therapies. It has shown potential in preclinical and clinical trials for various cancers, including melanoma and lung cancer, highlighting its role in personalized cancer immunotherapy and expanding the horizons of targeted cancer treatment strategies. Still, studies describing the reactivation of tumor suppressor genes in cancer cells are rare. This study aimed to investigate whether this class of biological agents is suitable for reactivating tumor suppressor genes in HGSOC. Here, the vector-based expression of DAPK1-clone 6 has been shown to trigger apoptosis in ovarian cancer lines and sensitize cancer cells to chemotherapeutics, the mainstays of ovarian cancer therapy. Clone 6 represents a truncated form of DAPK1 that encompasses (i) the kinase domain of DAPK1 (KD), whose activity is crucial for the death-promoting potential of DAPK1, (ii) the ankyrin repeats domain (AR), which is involved in protein–protein interaction and is important for DAPK1’s ability to interact with other proteins. Ankyrin repeats help stabilize the structure of DAPK1 and facilitate its interactions with various substrates and regulatory proteins. (iii) The death domain mediates protein–protein interaction that lead to apoptotic signaling. To investigate the reactivation of tumor suppressors via mRNA-based delivery, we utilized an in vitro transcription (IVT) system driven by a T7 RNA Polymerase promoter in a bacterial vector to generate truncated human DAPK1 mRNAs. This approach allowed us to evaluate the translational efficiency and mRNA stability systematically [38]. The IVT-derived Flag-tagged truncated DAPK1 (Clone 6) was designated as ΔDAPK1-mRNA for subsequent studies.

Liposomes enhance drug delivery by improving stability, reducing toxicity, and increasing the accumulation of drugs in tumor tissues. This targeted approach improves therapeutic efficacy and minimizes adverse effects, offering promising advancements in ovarian cancer therapy [39]. Thus, we applied a liposomal system using Lipofectamine Messenger MAX Transfection Reagent to deliver ΔDAPK1-mRNA to HGSOC cells. Our goal was to reactivate specific DAPK1 functions by using the ΔDAPK1-mRNA transfection of HGSOC cell lines and primary tumor cells.

**Figure 8:**
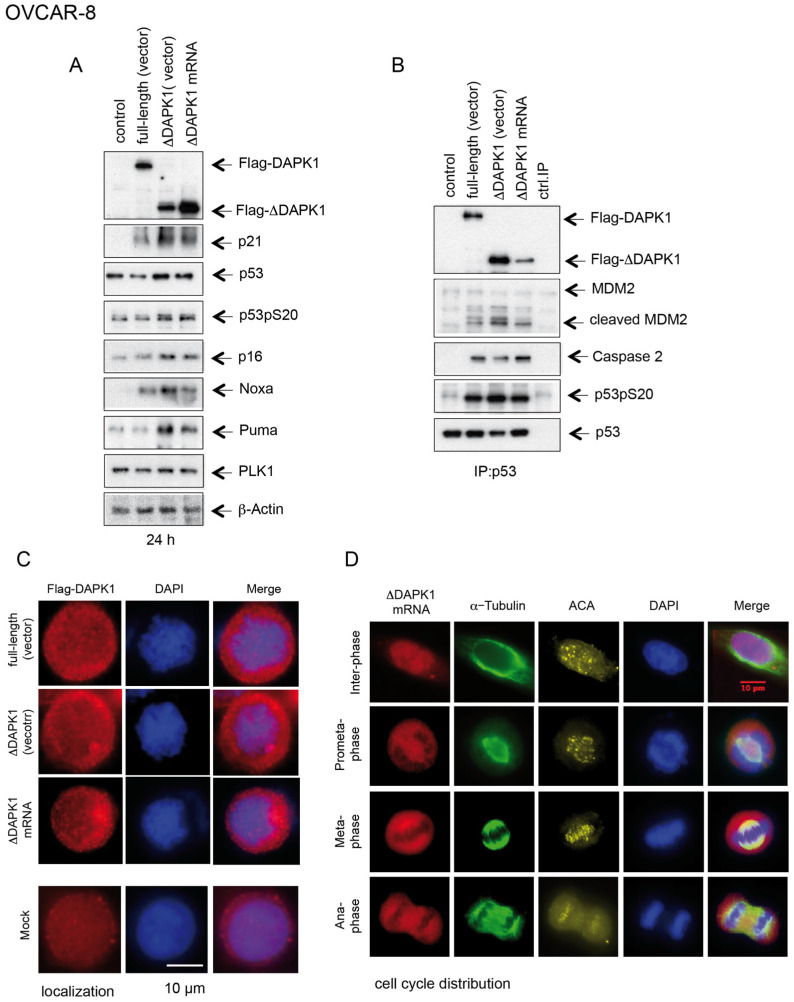
Impact of reactivation of DAPK1 functions in HGSOC cell lines by ΔDAPK1-IVT mRNA or by vector-based expression of full-length DAPK1 on p53 signaling and apoptosis. (**A**) Using Flag, p21, p53, p53pS20, p16, Noxa, Puma, PLK1, p14ARF, and β-Actin antibodies, cell lysates were subjected to WB. (**B**) An immunoprecipitation with p53-specific antibodies based on cell lysates shown in (**A**) was submitted to WB using Flag, MDM2, cleaved MDM2, Caspase-2, p53pS20, and p53 antibodies. (**C**) Interphase localization of ∆DAPK1: OVCAR-8 cells expressing either vector-based ∆DAPK1 or transfected with ∆DAPK1-IVT mRNA were fixed and processed for immunofluorescence using Flag antibodies, with DNA stained using DAPI. Both vector-expressed ∆DAPK1 and mRNA-derived ∆DAPK1 exhibited similar cytoplasmic localization (scale bar = 10 µm). (**D**) Mitotic localization of ∆DAPK1: OVCAR-8 cells expressing ∆DAPK1-IVT mRNA were synchronized into mitosis using nocodazole. After washing off nocodazole, cells were released for 2 h, then fixed and processed for immunofluorescence using Flag and α-tubulin antibodies. The truncated ∆DAPK1 form was observed in the cytoplasm, associating with the mitotic spindle during early mitosis (scale bar = 10 µm). (The uncropped blots are shown in Figures S11 and S12).

Functional analysis comparing full-length DAPK1 with truncated ΔDAPK1 variants (vector-based expression or delivered with IVT-mRNA) showed that constructs lacking the CaM autoregulatory domain generated stronger p53-Ser20 phosphorylation and a greater induction of p21, Noxa, and Puma versus wt full-length DAPK1 (Figure 8A). However, p14ARF expression was similarly upregulated following the delivery of wt or the truncated version of DAPK1 (Figure 8A). Previous studies have demonstrated that the PIDD-dependent activation of Caspase-2 results in the cleavage of the RING domain of MDM2, the region directly responsible for ubiquitinating p53. Despite this cleavage, the truncated form of MDM2 retains its ability to bind p53, thereby enhancing its stability [40]. MDM2 could bind p53 in our IP experiments across all three clones (full-length and truncated forms) and, to a lesser extent, in the control untreated group (Figure 8B). However, in full-length and ΔDAPK1-expressing groups (vector- and IVT-mRNA-based expression), we observed the presence of both the uncleaved and cleaved forms of MDM2 in the precipitates, with high levels of cleaved MDM2 associated with p53 and Caspase-2. This suggests that the full-length and truncated ΔDAPK1 promote the Caspase-2-dependent cleavage of MDM2, thereby preventing p53 degradation and supporting its stabilization (Figure 8B).

The immunofluorescence study of full-length DAPK1 and ΔDAPK1 (both expressed from recombinant vectors) and ΔDAPK1 (transfected IVT mRNA) in interphase cells showed that all three proteins are located in the cytoplasm (Figure 8C), consistent with DAPK1’s role in regulating cell death and autophagy [41]. During mitosis, ΔDAPK1 encoded by the transfected IVT-mRNA remained localized in the cytoplasm of dividing cells. However, we observed that the truncated form interacted with the mitotic spindle from prometaphase to metaphase and was present at the spindle midzone during anaphase (Figure 8D). In contrast, our previous study demonstrated that the endogenous full-length DAPK protein is strongly associated with the centrosomes during the early stages of mitosis and is not detected at the spindle midzone during anaphase [17]. This suggests that while the truncation of DAPK does not affect its interphase localization, it may disrupt its proper localization during mitosis. Further investigation is required to understand the consequences of this mislocalization during cell division.

**Figure 9:**
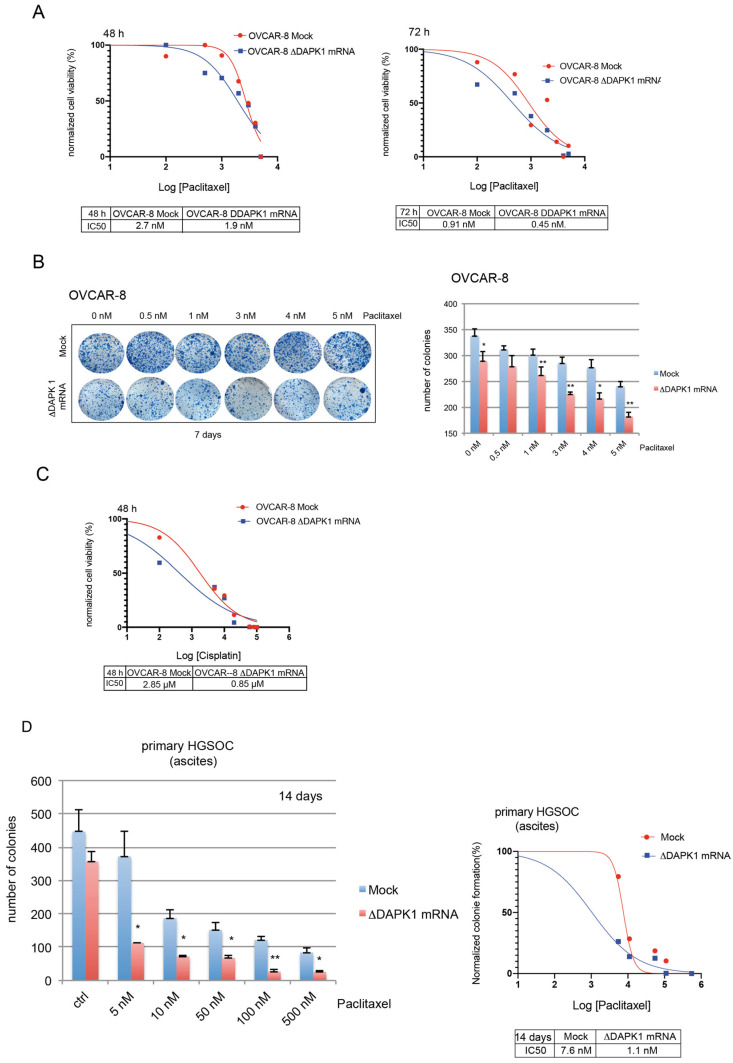
Effects of DAPK1 reactivation by ΔDAPK1-IVT mRNA on the survival of HGSOC cells. OVCAR-8 cells (mock) and OVCAR-8 cells transfected with ΔDAPK1-IVT mRNA were treated with increasing concentrations of Paclitaxel, and their proliferation was measured using a Cell Titer-Blue Cell Viability assay throughout 72 h, with measurements taken after 48 h (**left panel**) (**A**) and 72 h (**right panel**) (**A**). The proliferation rates of the treated cells, an indicator of cell viability, were normalized against their respective DMSO-treated counterparts (vehicle control  =  0 on the *x*-axis) and subsequently used to calculate the IC_50_ values of each cell type. The plots represent the IC_50_ values at 48 and 72 h. (**B**) On day 7, the number of 3D colonies (dependent on dosage) of OVCAR-8 cells transfected with ΔDAPK1-IVT mRNA, compared to control cells in the **left panel** of the representative photos. A bar graph illustrates the dose-dependent distribution of colonies (**right panel**; *n* = 3). Unpaired, two-tailed Student’s *t*-test with * *p* < 0.05 and ** *p* < 0.01. (**C**) Mock-treated OVCAR-8 cells and OVCAR-8 cells transfected with ΔDAPK1-IVT mRNA were treated with increasing concentrations of Cisplatin. Their proliferation and the IC_50_ values were measured as described for (**A**). (**D**) Cancer cells isolated from HGSOC ascites transfected with ΔDAPK1-IVT mRNA were treated with increasing concentrations of Paclitaxel. On day 14, the dose-dependent number of 3D colonies was depicted as a bar graph (**left panel**; *n* = 3). Unpaired, two-tailed Student’s *t*-test with * *p* < 0.05 and ** *p* < 0.01. The colony numbers were normalized with their respective DMSO-treated counterparts and used to calculate the IC_50_ (**right panel**).

Previous experiments about the impact of vector-based expression on the response to standard therapeutics had demonstrated that the reactivation of DAPK1 sensitizes ovarian cancer cells to Paclitaxel and Cisplatin (Figure 6 and Figure 7). Next, we tested the impact of IVT ΔDAPK1-mRNA on the viability of ovarian cancer cells. The expression of the activated form of DAPK1, achieved by transfecting IVT ΔDAPK1 mRNA, significantly increased the sensitivity of OVCAR-8 cells to Paclitaxel (Figure 9A). The clonogenic assay supported the sensitizing activity of IVT ΔDAPK1-mRNA to OVCAR-8 cells treated with Paclitaxel (Figure 9B). The ability of IVT ΔDAPK1-mRNA to sensitize OVCAR-8 cells to Cisplatin treatment (Figure 9C) confirmed earlier findings observed with vector-based expression (Figure 7C,D). Furthermore, transfection with ΔDAPK1-mRNA reduced the colony-forming capacity and enhanced Paclitaxel sensitivity in primary tumor HGSOC cells derived from patient ascites (Figure 9D). Additional experiments using OVCAR-3 cells treated with Paclitaxel or Cisplatin confirmed the sensitization by the expression of ΔDAPK1 mRNA (Figure S3A,B), supporting our previous data using the vector-based expression of ΔDAPK1.

### 3.7. IVT ΔDAPK1-mRNA Triggers Apoptosis and Suppresses Tumor Growth in HGSOC 3D Model

**Figure 10:**
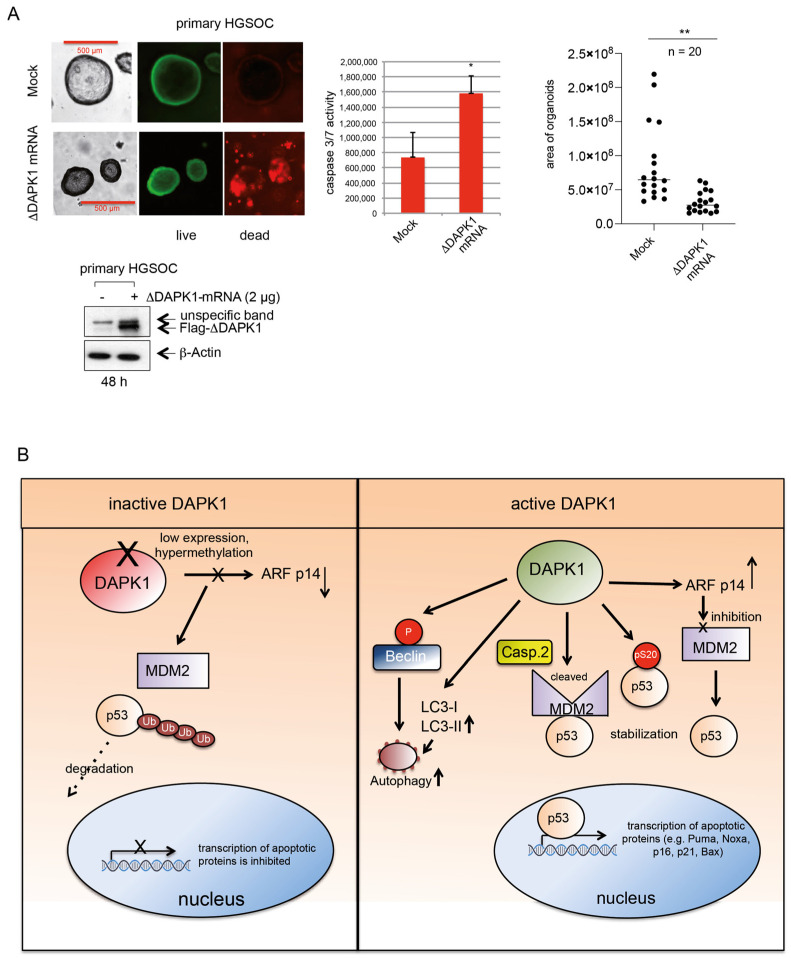
Effects of DAPK1 reactivation on the survival of primary HGSOC cells. (**A**) Patient-derived organoids were transfected with 2 μg of ΔDAPK1-IVT mRNA. On day 2, following transfection with 2 μg of ΔDAPK1-IVT mRNA, HGSOC organoids are depicted in representative photos (**left panels**), and a live/dead assay was performed. Flag and β-actin antibodies were used to perform WB on the lysates of HGSOC organoids mock or transfected with ΔDAPK1-IVT mRNA (**lower left panel**). The Caspase-Glo 3/7 assay was used to analyze mock- or ΔDAPK1-IVT mRNA-transfected HGSOC organoids (**right panel**; *n* = 3). * *p* < 0.05; unpaired, two-tailed Student’s *t*-test. ImageJ 1.0 was used to calculate the size of the organoids (**middle panel**; *n* = 20). ** unpaired, two-tailed Student’s *t*-test; *p* < 0.01. (**B**) Targeted reactivation of DAPK1 in ovarian cancer cells and resulting changes in cellular signaling. Ovarian cancer lacking DAPK1 exhibits a reduced expression of p14ARF, enabling MDM2 to stably bind to p53, resulting in its ubiquitination and degradation. This degradation impairs the transcription of p53-dependent apoptotic effectors and cell cycle regulators, promoting cancer cell proliferation and survival. In contrast, the reactivation of DAPK1 using an IVT-mRNA triggers multiple pathways that stabilize and activate p53, promoting cell cycle arrest and triggering cell death through the p53-dependent transcription of apoptosis and cell cycle effectors. Furthermore, active DAPK1 promotes Caspase-2 activation, which targets MDM2 for cleavage, thus preventing p53 degradation. Additionally, DAPK1 phosphorylates p53 at S20, further enhancing its activity. Reactivated DAPK1 also indirectly increases p14ARF expression, stabilizing p53 and facilitating the transcriptional activation of apoptosis and cell cycle regulatory genes. Finally, DAPK1 promotes the conversion of LC3A/B-I to LC3A/B-II, and both processes are crucial in inducing autophagy. Hence, the vector-based and IVT-mRNA-dependent reactivation of DAPK1 demonstrates a dual role in promoting p53 stability and autophagy, emphasizing its therapeutic potential in targeting ovarian cancer.

To evaluate the therapeutic potential of DAPK1 restoration on patient-derived cancer cells, we transfected primary HGSOC cells with IVT ΔDAPK1 mRNA. Live/death assays and Caspase 3/7 activity measurements in patient-derived organoids demonstrated that ΔDAPK1-mRNA treatment significantly induced cell death (Figure 10A, left and middle panels). Furthermore, ΔDAPK1-mRNA treatment significantly reduced PDO growth, confirming its potent pro-apoptotic effects in HGSOC (Figure 10A, right panel).

## 4. Discussion

A perfect balance of active and quiet genes governs an organ’s appropriate growth and maturation, which is particularly important during delicate cellular processes, such as embryonic development, X-chromosome inactivation, and genomic imprinting [42]. On the other hand, gene expression can be altered to promote cancer by aberrant DNA methylation and structural chromatin modifications [43]. Genes involved in DNA repair, apoptosis, differentiation, drug resistance, angiogenesis, and metastasis can have their expression decreased due to DNA methylation, which creates a tightly knit and restrictive chromatin structure. The hypermethylation of specific gene promoters has been identified in ovarian cancers [44]. The promoter hypermethylation of tumor suppressors, *BRCA1* and *RASSF1A,* was significantly higher in ovarian cancers compared to non-neoplastic tissues [45]. In this study, we identified the aberrant expression of *DAPK1* in HGSOC. The downregulation of *DAPK1* in cancer tissues compared to their benign counterparts was shown to be correlated with a poor prognosis in the early stages of the disease and was found to originate from promoter methylation. Thus, we added DAPK1, a new gene, to the list of hypermethylated tumor suppressors, including *BRCA1* and *RASSF1A*, that are prognosis-relevant in ovarian cancer.

The primary method of inactivating some tumor suppressor genes, including the traditional tumor suppressor p53, is mutation. The p53 gene is frequently mutated or inactivated in cancers [46]. In HGSOC, the frequency of p53 mutations is particularly high, at 95%. This is the highest rate compared to the mutational rate of p53 in all other human tumors. Reactivating the wild-type activity of mutant p53 is a useful strategy for halting tumor growth [47,48]. Numerous investigations have revealed that peptide medications and small molecules, such as CP-31398 [49], RITA [50], PEITC [51], NSC319726 [52], Chetomin [53], ReACp53 [54], and pCAPs [55], can cause modifications in the spatial conformation and folding pattern of mutant p53. Clinical studies are currently being conducted for APR-246, COT1-2, PC14586, and arsenic trioxide (ATO). Accordingly, the data indicate that restoring mutant p53 to its wild-type conformation represents a viable anticancer strategy.

Nevertheless, drugs for restoring p53 function have not yet been approved for treating cancer patients. Thus, in particular, for treating patients suffering from aggressive HGSOC, alternative strategies and targets are urgently needed to reactivate p53 functions. Therapeutic mRNAs hold significant potential in modern medicine, offering a novel approach to treating various diseases. Prior to the use of mRNA, a mammalian vector system was employed in our investigation, and DAPK1 was exogenously expressed in HGSOC cell lines to reactivate DAPK1 functions. Additionally, liposomes were used to transfer IVT-DAPK1 mRNA into different 3D HGSOC model systems, including cancer cell lines and patient-derived organoids. The cells were then monitored for proliferation, apoptosis, colony formation ability, and cell cycle progression. The introduction of full-length DAPK1 induced the potent inhibition of HGSOC, cell proliferation, dose-dependent apoptosis, and regulated autophagy. In our study, we have significantly shortened the open reading frame of wt DAPK1 from 1430 amino acids to 697 amino acids in ΔDAPK1 (clone 6), which contains only the kinase domain (KD), the ankyrin repeats (AR), and the death domain (DD). The primary reason for shortening the wt open reading frame was to enhance transfection and translation efficiency for preclinical and later clinical trials involving therapeutic mRNA. The N-terminal end of full-length DAPK1 contains a conventional kinase domain and a Ca^2+^/CaM autoregulatory region that, in the absence of CaM, interacts with the catalytic cleft to decrease enzymatic activity and prevents contact with external substrates. While the KD of DAPK1 was preserved in ΔDAPK1, its Ca^2+^/CaM autoregulatory region was deleted to create an apoptotic form of the kinase containing the death domain (DD) that is constitutively active. We demonstrated that the exogenous expression of full-length DAPK1 and its truncated form, ΔDAPK1, can significantly increase the phosphorylation of p53S20. Still, the direct comparison confirmed our hypothesis that the truncated form of ΔDAPK1 has higher kinase activity compared to full-length DAPK, based on the phosphorylation level of its substrate p53S20. In addition to KD and DD, ΔDAPK1 encompasses the ankyrin repeats (AR), which facilitate protein–protein interaction and promote the ability of ΔDAPK1 to recognize the endogenous substrates of wt full-length DAPK1.

To stabilize p53 in response to genotoxic stress, kinases such as ATM (Ataxia-Telangiectasia Mutated) and CHK2 (Checkpoint Kinase 2) specifically target Serine 20 for phosphorylation [56]. This phosphorylation disrupts the interaction between MDM2 and p53, lowering the MDM2-dependent ubiquitination of p53 and leading to its stabilization [57]. Consequently, the accumulation of p53 triggers the expression of genes regulating cell cycle progression, apoptosis, and DNA repair pathways [58]. Upon the release of p53 from MDM2, this later retains its E3 ubiquitin ligase activity and can target other proteins, albeit less efficiently, without p53. The feedback loop p53-MDM2 may also trigger MDM2 autoubiquitination and degradation, reducing its levels [59]. Caspase-2 is activated by the p53 target gene PIDD within the PIDDosome complex [40], which includes the adaptor protein RAIDD/CRADD [60,61]. Upon activation, Caspase-2 cleaves MDM2, removing its RING domain and impeding p53 ubiquitination and degradation. Thus, the PIDD-Caspase-2-MDM2 axis appears to be crucial for p53 stabilization, as verified in our immunoprecipitation assays. This experiment revealed the interactions between cleaved MDM2 products and Caspase-2, as well as stable p53, following the restoration of DAPK1 or ΔDAPK1 expression using vector-based or IVT mRNA delivery. Thus, the expression of ΔDAPK1 directly stabilizes p53 through Ser20 phosphorylation and facilitates the Caspase-2-dependent cleavage of MDM2, reinforcing p53 functionality.

The human alternate reading frame protein (p14ARF) has been shown to interact with MDM2, interfering with its binding to p53 and consequently stabilizing p53 [62]. Evidence suggests that DAPK1 may activate the p53 pathway via p19 ARF, the murine homolog of human p14ARF, although the exact mechanism remains unclear [11]. Consistent with this, restoring ΔDAPK1 expression through IVT-mRNA in OVCAR-8 cells resulted in increased p14ARF levels, coinciding with the enhanced stabilization of p53. Concomitant with this, our Western blot analysis further confirmed that p14ARF and critical p53 targets, such as p21, Noxa, and Puma, were significantly increased in OVCAR-8, suggesting the activation of p53 in ovarian cancer cells treated with vector-based or IVT-mRNA-delivered ΔDAPK1 (Figure 10B).

LC3 is a key protein for autophagosome formation and maturation [32]. LC3 exists in two distinct forms: LC3-I, the cytosolic form, and LC3-II, the lipidated form that associates with autophagosome membranes [32]. The kinase-mediated phosphorylation of LC3 has been shown to facilitate the transition of LC3-I to the lipidated, autophagosome-associated LC3-II, a process essential for autophagosome elongation and closure, thereby regulating autophagy [33]. Previous research indicated that this conversion is decreased in DAPK1-deficient cells, suggesting that DAPK1 is critical for the transition to LC3-II, possibly through indirect mechanisms [63]. Consistent with these findings, we observed enhanced levels of autophagosome-associated LC3 A/B-II in our assays, suggesting the increased autophagic activity in ovarian cancer cells after the mammalian vector-dependent restoration of ΔDAPK1 (Figure 10B).

While our findings demonstrate the therapeutic potential of DAPK1 reactivation in HGSOC, several limitations should be acknowledged: 1—Our in vitro and 3D organoid models, though informative, may not fully recapitulate the tumor microenvironment or immune interactions observed in vivo. 2—The efficacy of IVT-mRNA delivery was primarily evaluated in cell lines and patient-derived organoids; translational studies in animal models are needed to assess the pharmacokinetics and potential off-target effects. Finally, clinical applicability may be limited by tumor heterogeneity and variable epigenetic landscapes in HGSOC patients, which could impact the efficacy of DAPK1 restoration. Addressing these limitations in future studies will strengthen the translational relevance of our approach.

Taken together, while multiple approaches are currently being tested in preclinical and clinical trials for the reactivation of p53 in cancer cells [49,50,51,52,53,54,55,64,65], drugs for the reactivation of p53 have not been approved yet for treating cancer patients. By overexpressing an upstream regulator of p53, such as DAPK1 or ΔDAPK1, we introduce a promising new strategy for restoring p53 functions, which could be a valuable future tool to enhance the armament against HGSOC. Our findings underscore the dual role of DAPK1 in regulating p53-dependent apoptosis and autophagy while also highlighting its potential as both a prognostic marker and therapeutic target for HGSOC. Our observations provide critical mechanistic insights into how DAPK1 influences ovarian cancer progression.

## 5. Conclusions

High-grade serous ovarian cancer treatment is limited by chemoresistance and frequent *p53* mutations. Our study demonstrates that restoring the tumor suppressor DAPK1, frequently downregulated in HGSOC, effectively induces apoptosis and reverses chemoresistance. An analysis of 1436 patient samples revealed DAPK1’s prognostic value in early-stage diseases of HGSOC. Functional studies showed that both full-length and truncated DAPK1 variants (∆DAPK1) triggered Caspase-dependent apoptosis, phosphorylated p53Ser20, and stabilized p53 via MDM2 cleavage in HGSOC cell lines. Remarkably, ∆DAPK1 reactivation sensitized cancer cells and patient-derived primary HGSOC (ascites) cells to Paclitaxel (two-fold IC_50_ reduction) and Cisplatin. These findings establish DAPK1 reactivation as a promising strategy to overcome chemoresistance in HGSOC, with truncated variants offering clinical advantages for therapeutic development. The dual p53-dependent and -independent mechanisms provide robust tumor suppression even in a p53-mutant background. Clinically, this highlights the potential of DAPK1-based therapies to enhance the efficacy of standard chemotherapy, particularly for patients with chemoresistant or p53-deficient tumors. Future research should focus on validating these effects in larger patient cohorts, optimizing delivery strategies for ∆DAPK1 variants, and evaluating the synergistic effects of these variants with emerging targeted therapies in clinical trials.

## Figures and Tables

**Table 1:** Aggregate clinical data for all patients and for stage 1+2 patients.

	All Patients	Stage 1+2 Patients
Average PFS follow-up	24.9 months	35.4 months
**Stage**		
1	96	96
2	67	67
3	919	-
4	162	-
**Subtypes**		
serous	1104	99
endometrioid	51	28
clear cell	31	24
**Grade**		
1	37	16
2	256	42
3	837	64
4	19	0
**Debulk**		
optimal	697	98
suboptimal	459	7
**Chemotherapy**		
Yes	1277	94
No	159	69

## Data Availability

The data presented in this study are openly available on the TCGA platform [OV-TCGA] [https://cancergenome.nih.gov, accessed on 1 August 2024] [cg22571217].

## Supplementary Materials

The following supporting information can be downloaded at: https://www.mdpi.com/article/10.3390/cancers17121910/s1, Figure S1. Effects of DAPK1 re-activation by vector-based DAPK1 expression on the survival of HGSOC cells. (A) OVCAR-4 cells or (B) OVCAR-8 cells transfected with a vector expressing full-length DAPK1 were treated with increasing concentrations of Paclitaxel. The cell proliferation was measured using an CellTiter-Blue Cell Viability assay over a period of 72 h, with measurements taken at 48 h or 72 h. The proliferation rates of the treated cells, an indication of cell-viability, were normalized with their respective DMSO-treated counterparts (vehicle control = 0 on the x-axis) and subsequently used to calculate the IC_50_ values of each cell type. The plots represent the IC_50_ value at the 72 h-time point for OVCAR-4 (A), and at 48 h for OVCAR-8 (B). Figure S2. Impact of vector-based and stable transfected full-length DAPK1 on the survival of HGSOC cells under Paclitaxel treatment. (A) OVCAR-5 cells or (B) OVCAR-8 cells stably transfected with a vector expressing full-length DAPK1 were treated with increasing concentrations of Paclitaxel. Lysates of stably expressing cells were subjected to SDS-PAGE and stained using antibodies for Flag-DAPK1. A Caspase-Glo 3/7 assay was used to measure the Caspase-3/7 activity of OVCAR-5 or (B) OVCAR-8, both stably expressing DAPK1, treated with increasing doses of Paclitaxel. *p* < 0.05 for *, *p* < 0.01 for **, and *p* < 0.001 for ***. Student’s two-tailed, unpaired *t*-test. Figure S3. Effects of DAPK1 re-activation by ΔDAPK1-IVT mRNA on the survival of HGSOC cells upon Paclitaxel treatment. (A) OVCAR-3 cells (mock) and OVCAR-3 cells transfected with ΔDAPK1-IVT mRNA were treated with increasing concentrations of Paclitaxel, and their proliferation was measured using an CellTiter-Blue Cell Viability assay over a period of 72 h, with measurements taken at 48 h and 72 h. The proliferation rates of the treated cells, an indication of cell viability, were normalized with their respective DMSO-treated counterparts (vehicle control = 0 on the x-axis) and subsequently used to calculate the IC_50_ values of each cell type. The plots represent the IC_50_ values at the 48 h and 72 h time points. (B) OVCAR-3 cells transfected with ΔDAPK1-IVT mRNA were treated with increasing concentrations of Cisplatin with measurements taken at 24 h. Their proliferation and the IC_50_ values were measured as described for (A). Figures S4–S13: Uncropped Blots.
