# Correction: Raab et al. Truncated DAPK Variants Restore Tumor Suppressor Activity and Synergize with Standard Therapies in High-Grade Serous Ovarian Cancer. *Cancers* 2025, *17*, 1910

**DOI:** 10.3390/cancers18142351

**Published:** 2026-07-21

**Authors:** Monika Raab, Khayal Gasimli, Balázs Győrffy, Samuel Peña-Llopis, Sven Becker, Mourad Sanhaji, Klaus Strebhardt

**Affiliations:** 1 Department of Gynecology, Medical School, Goethe-University, 60596 Frankfurt am Main, Germany; mraab@med.uni-frankfurt.de (M.R.); khayal.gasimli@unimedizin-ffm.de (K.G.); sven.becker@ukffm.de (S.B.); 2 Department of Bioinformatics, Semmelweis University, 1094 Budapest, Hungary; gyorffy.balazs@yahoo.com; 3 Department of Biophysics, Medical School, University of Pecs, 7624 Pecs, Hungary; 4 HUN-REN TTK Cancer Biomarker Research Group, 1117 Budapest, Hungary; 5 Translational Genomics, Department of Ophthalmology, University Hospital Essen, 45147 Essen, Germany; samuel.pena-llopis@uk-essen.de; 6 German Cancer Consortium (DKTK) at the University Hospital Essen and German Cancer Research Center (DKFZ), 69120 Heidelberg, Germany; 7 German Cancer Research Center (DKFZ), 69120 Heidelberg, Germany

In the original publication [1], an error occurred during the assembly of Figure 2E. The revised Figure 2E is now based on the correct assembly of the corresponding experimental data, and the original Western blot scans have been provided for transparency. The corrected Figure 2E is shown below.

The authors state that the scientific conclusions are unaffected. This correction was approved by the Academic Editor. The original publication has also been updated as well as the Supplementary Materials.

## Figures and Tables

**Figure 2:**
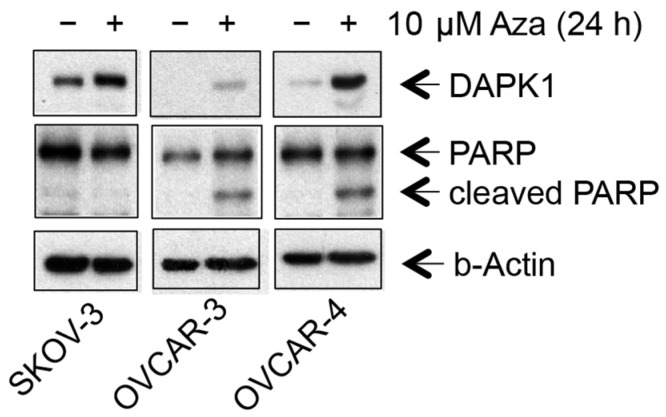
(**E**) Ovarian cancer cell lines were treated with 10 µM of 5-Azacytidine for 24 h. Cell lysates were immunoblotted for DAPK1, PARP, and β-Actin.
