# Supplementary material for: Truncated DAPK Variants Restore Tumor Suppressor Activity and Synergize with Standard Therapies in High-Grade Serous Ovarian Cancer
Source: Cancers (Basel). 2025 Jun 8;17(12):1910. doi: 10.3390/cancers17121910 (PMC12190691; doi:10.3390/cancers17121910)

A

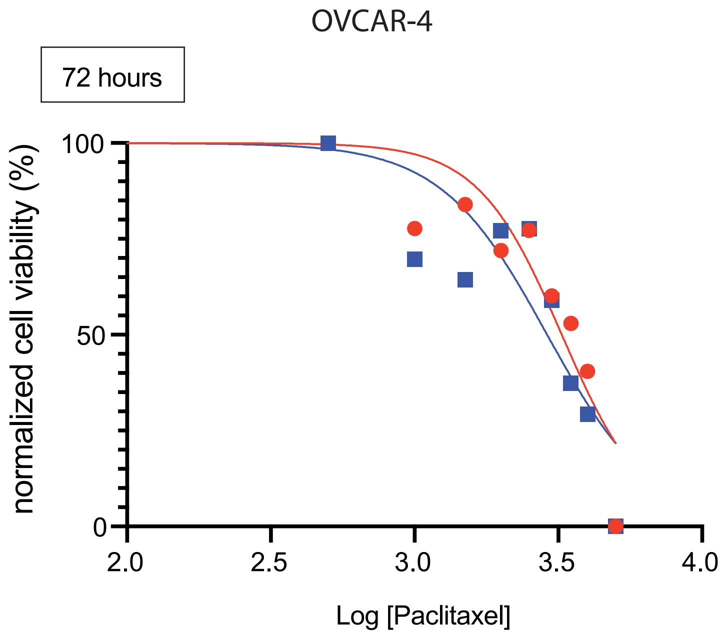

72 h	Mock	Flag-DAPK1
IC50	3.25 nM	2.89 nM

B

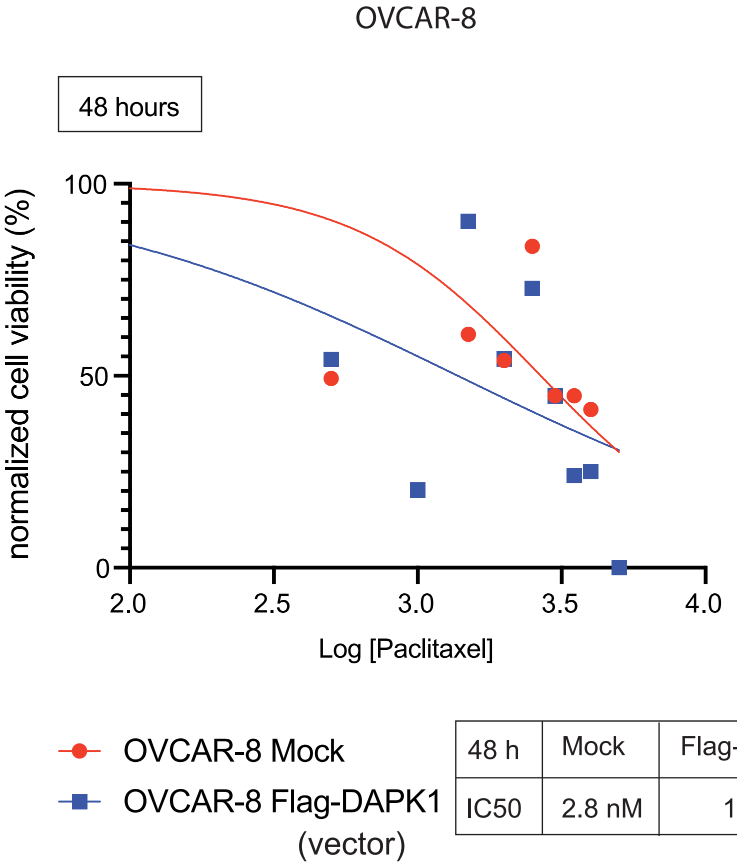

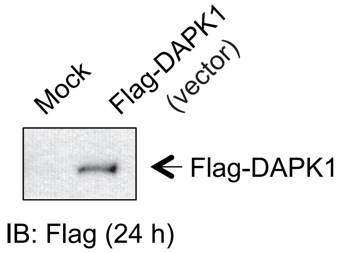

48 h	Mock	Flag-DAPK1
IC50	2.8 nM	1.4 nM

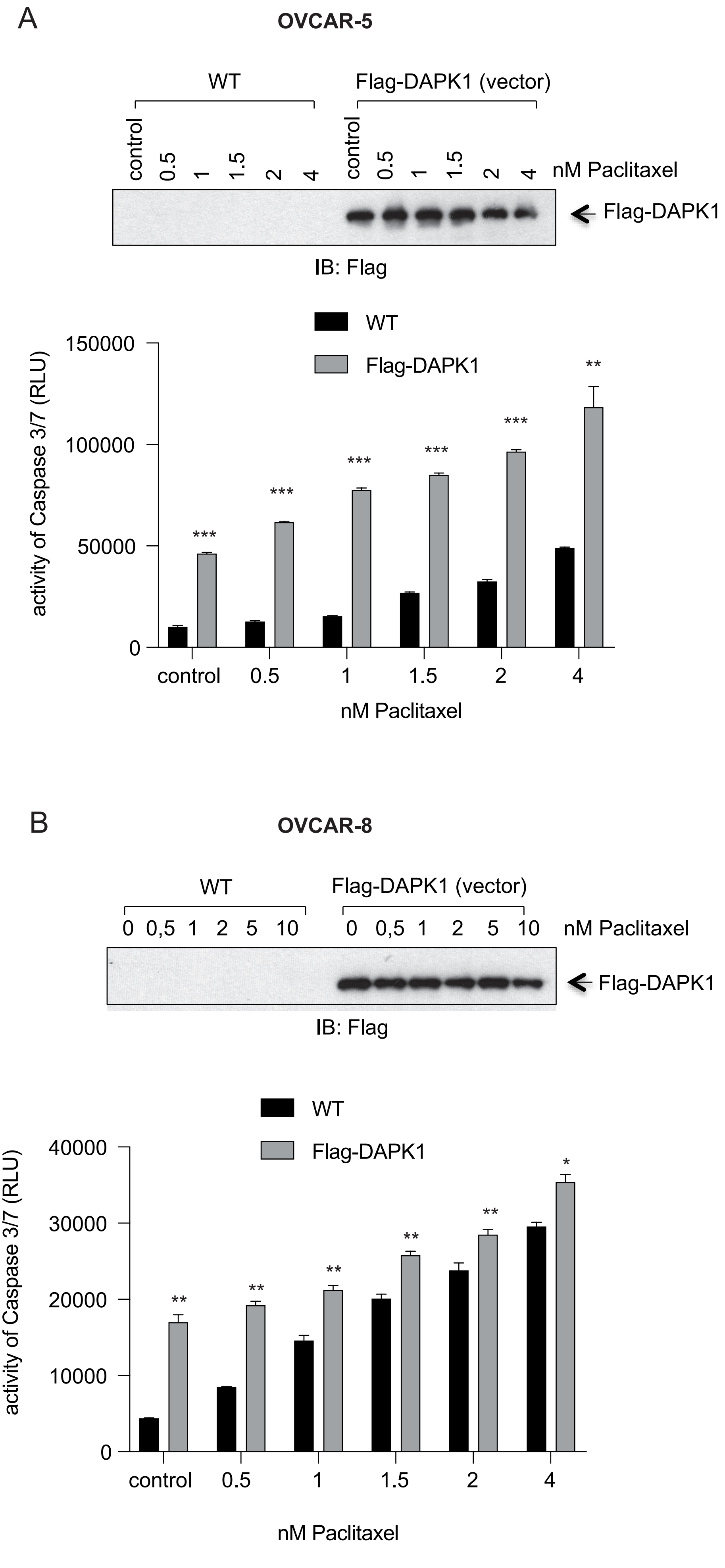
A
OVCAR-5
WT
Flag-DAPK1 (vector)
control 0.5 1 1.5 2 4 control 0.5 1 1.5 2 4 nM Paclitaxel
Flag-DAPK1
IB: Flag
WT
Flag-DAPK1
activity of Caspase 3/7 (RLU)
150000
100000
50000
0
***
**
control 0.5 1 1.5 2 4
nM Paclitaxel
B
OVCAR-8
WT
Flag-DAPK1 (vector)
0 0,5 1 2 5 10 0 0,5 1 2 5 10 nM Paclitaxel
Flag-DAPK1
IB: Flag
WT
Flag-DAPK1
activity of Caspase 3/7 (RLU)
40000
30000
20000
10000
0
**
*
control 0.5 1 1.5 2 4
nM Paclitaxel

Suppl. Fig. S3

A

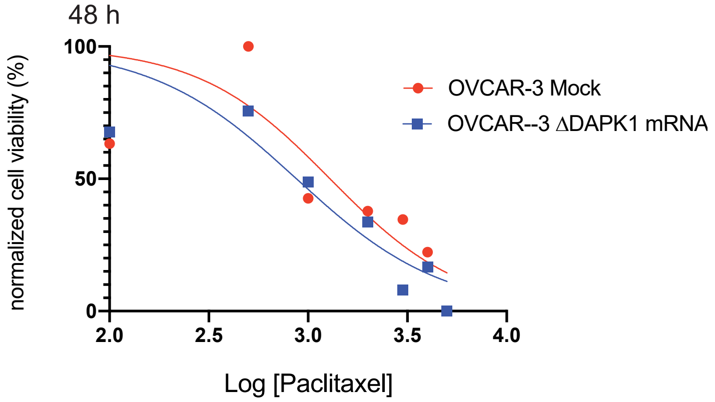

48 h	OVCAR-3 Mock	OVCAR-3 ΔDAPK1 mRNA
IC50	1.3 nM	0.87 nM

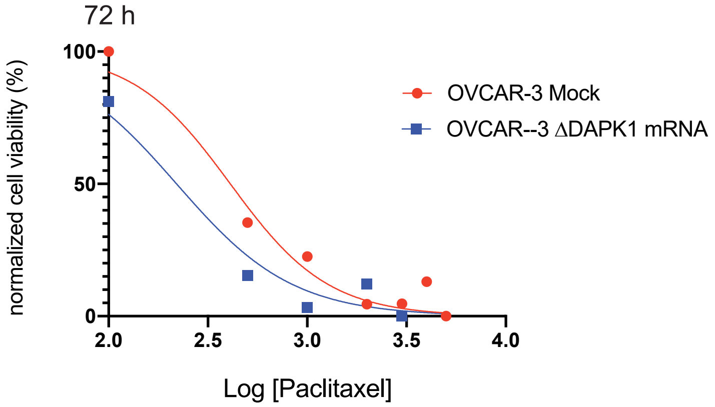

72 h	OVCAR-3 Mock	OVCAR-3 ΔDAPK1 mRNA
IC50	0.41 nM	0.21 nM

B

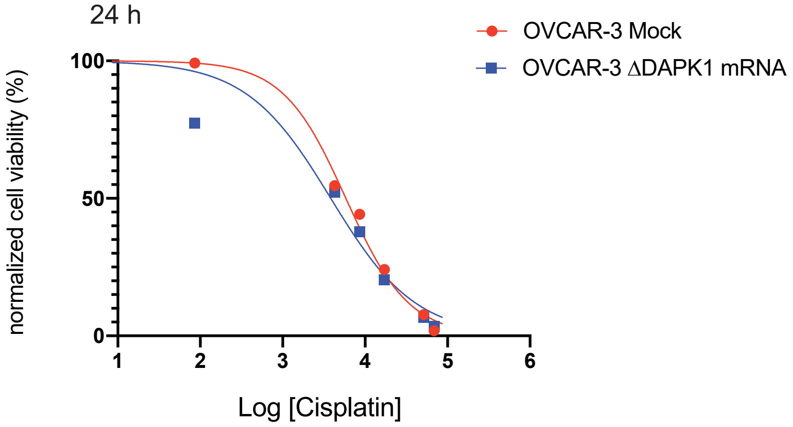

24 h	OVCAR-3 Mock	OVCAR-3 ΔDAPK1 mRNA
IC50	6.03 μM	1.86 μM

Figure 1A

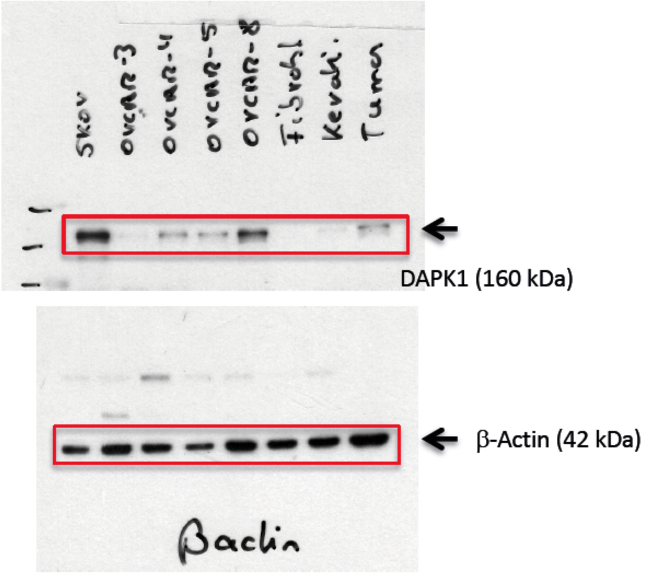

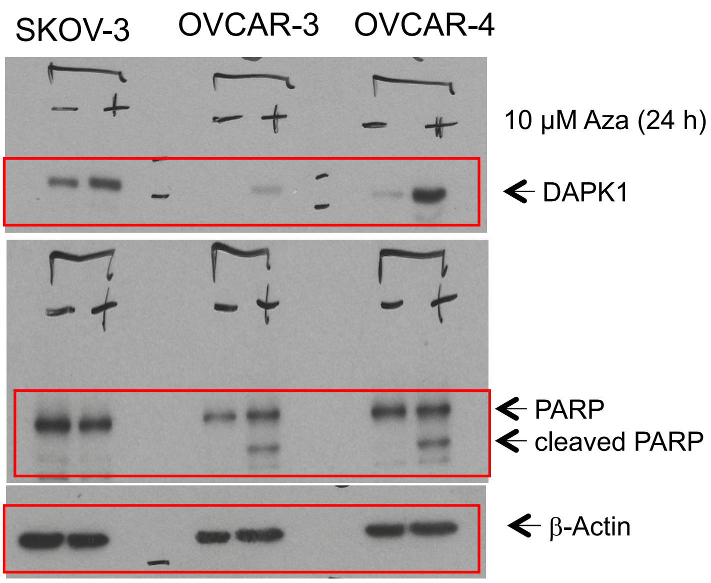

Figure 2E

Figure S4

Figure 3A

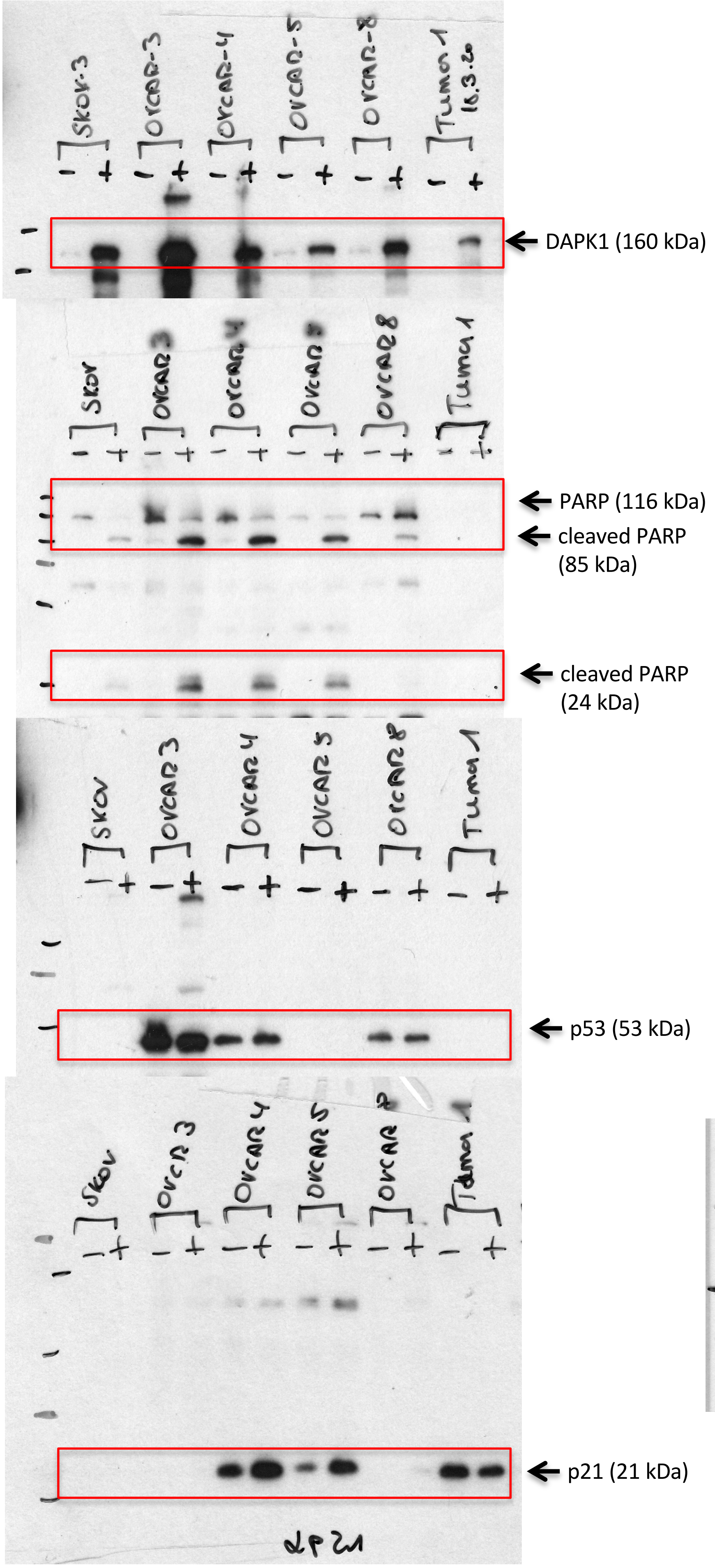

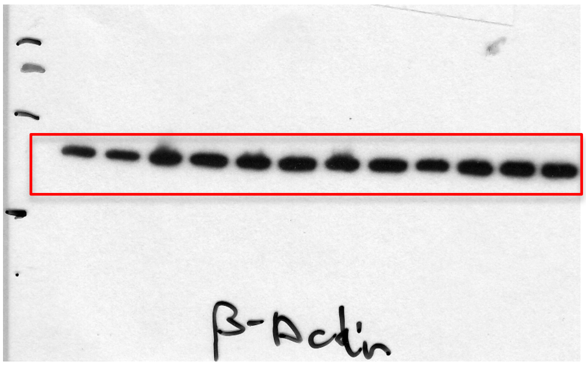

Figure S5

Figure 3C

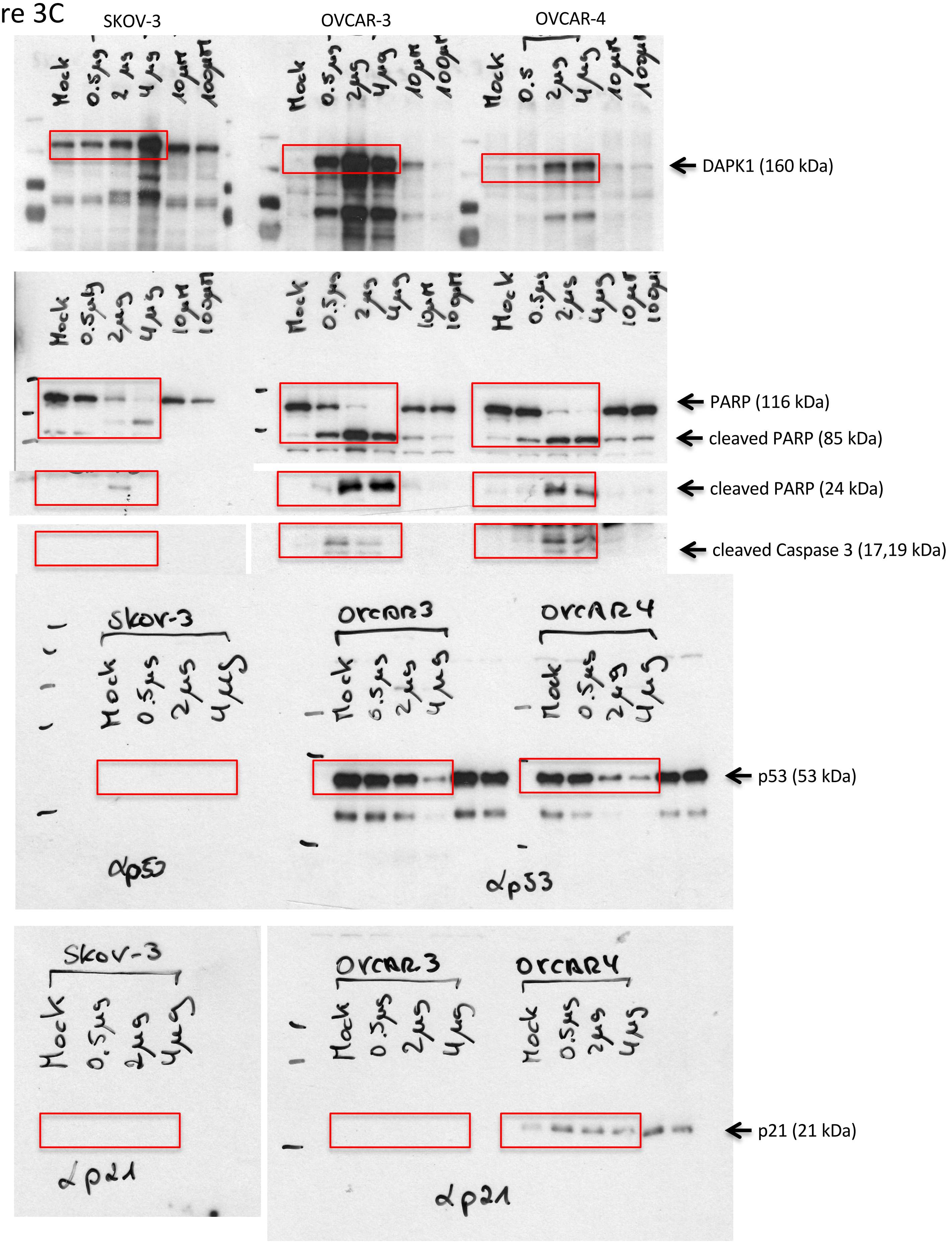

Figure S6

Figure 3C

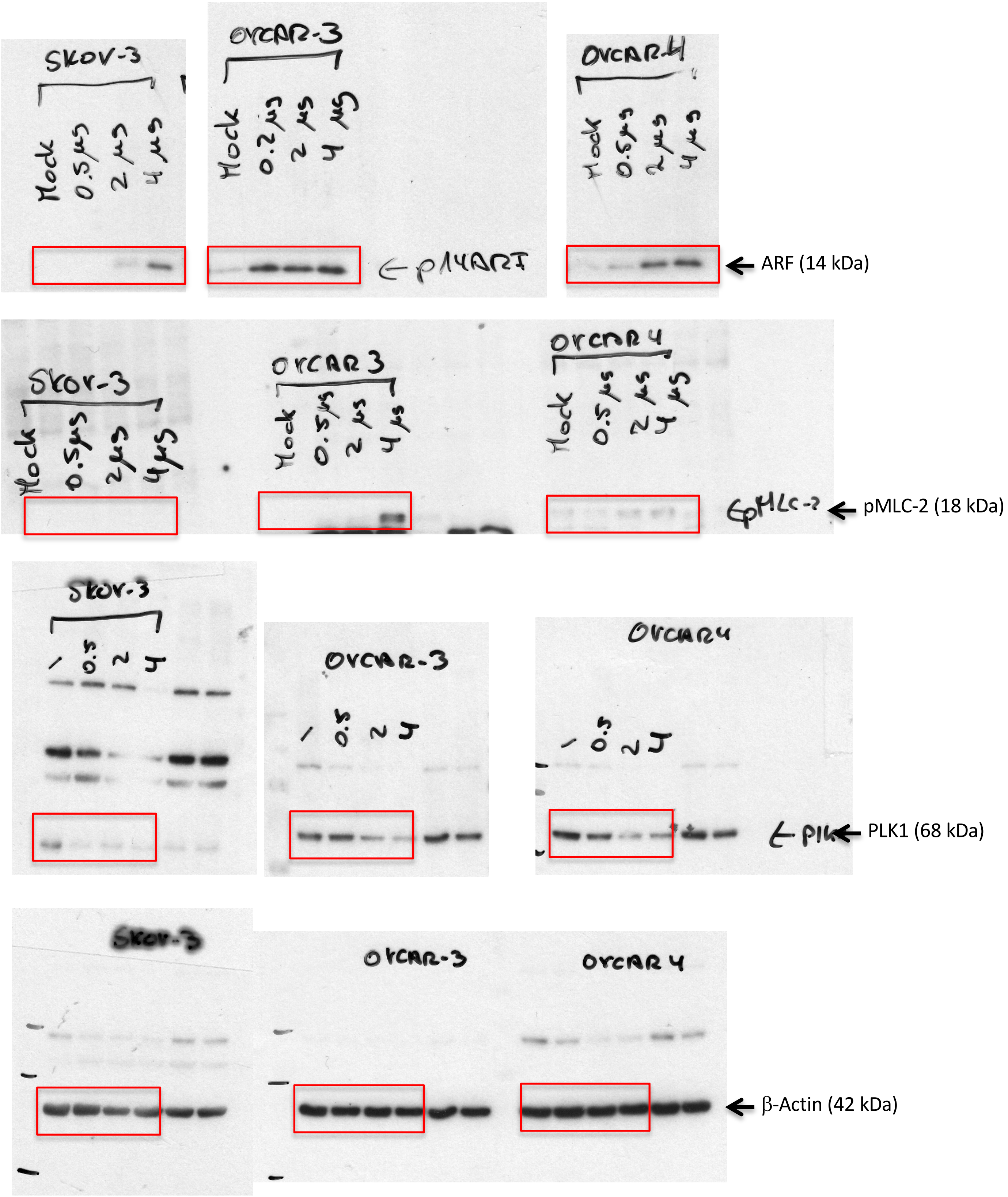

Figure S7

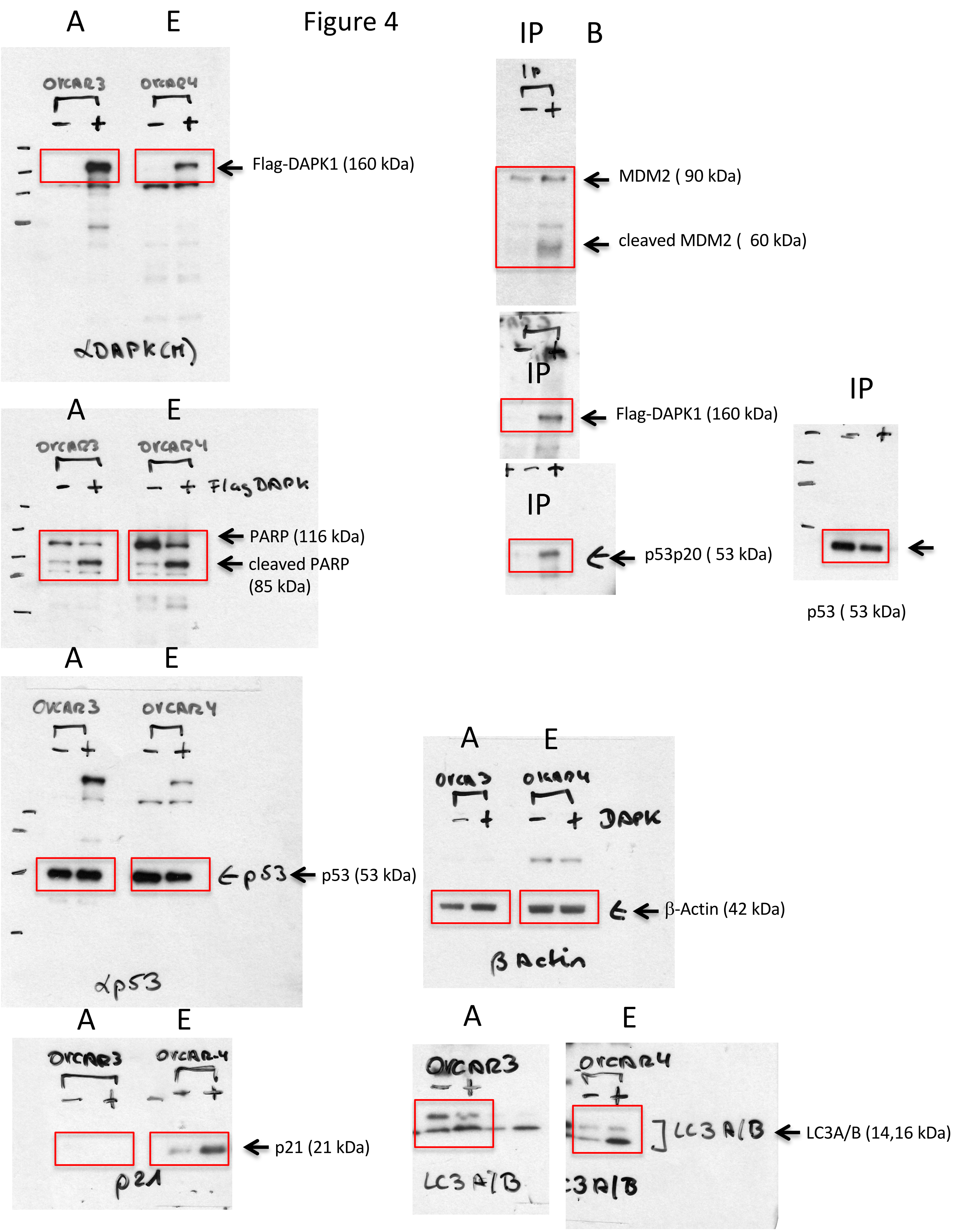
Figure 4
A
E
OVCAR3
OVCAR4
Flag-DAPK1 (160 kDa)
αDAPK(M)
IP
B
MDM2 (90 kDa)
cleaved MDM2 (60 kDa)
Flag-DAPK1 (160 kDa)
p53p20 (53 kDa)
p53 (53 kDa)
PARP (116 kDa)
cleaved PARP
(85 kDa)
FlagDAPK
p53 (53 kDa)
αp53
DAPK
β-Actin (42 kDa)
β Actin
p21 (21 kDa)
p21
LC3A/B (14,16 kDa)
LC3 A/B

Figure S8

Figure 5B

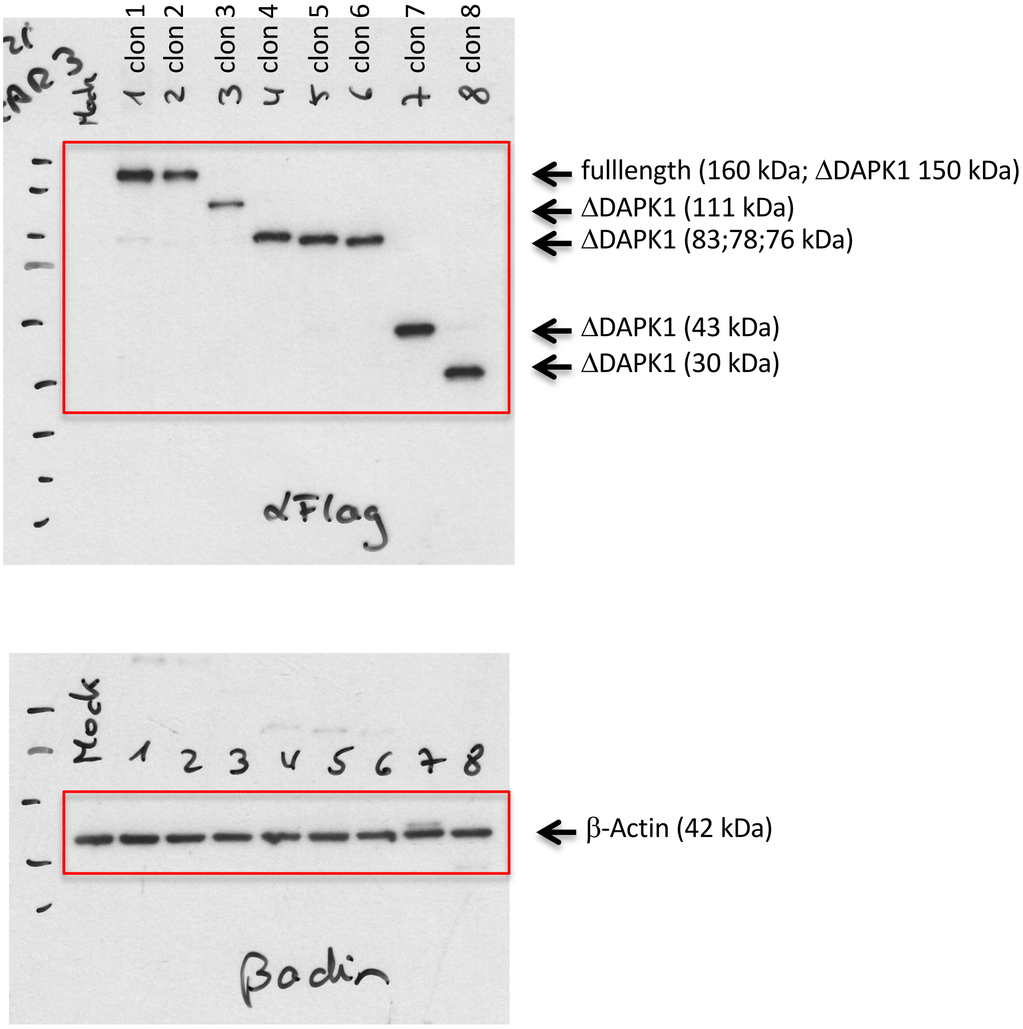

Figure S9

Figure 7A

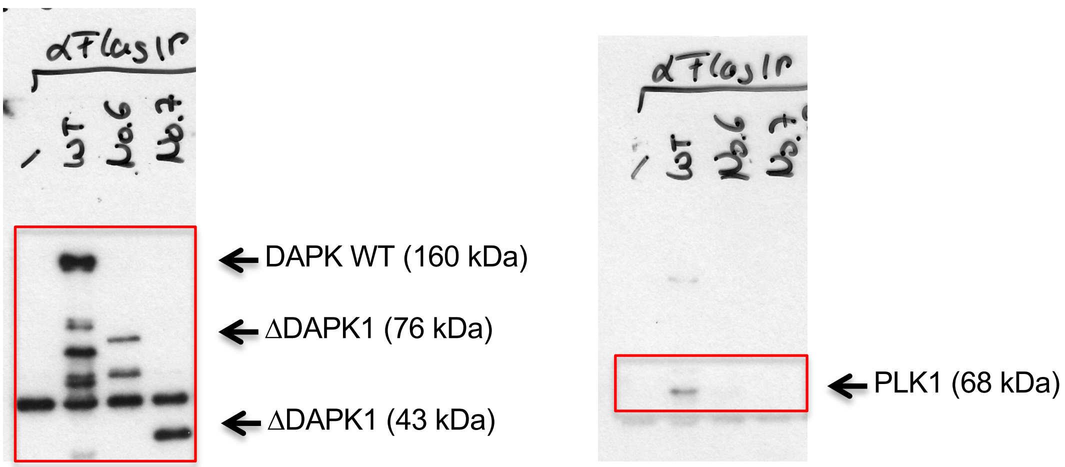

Figure 7D

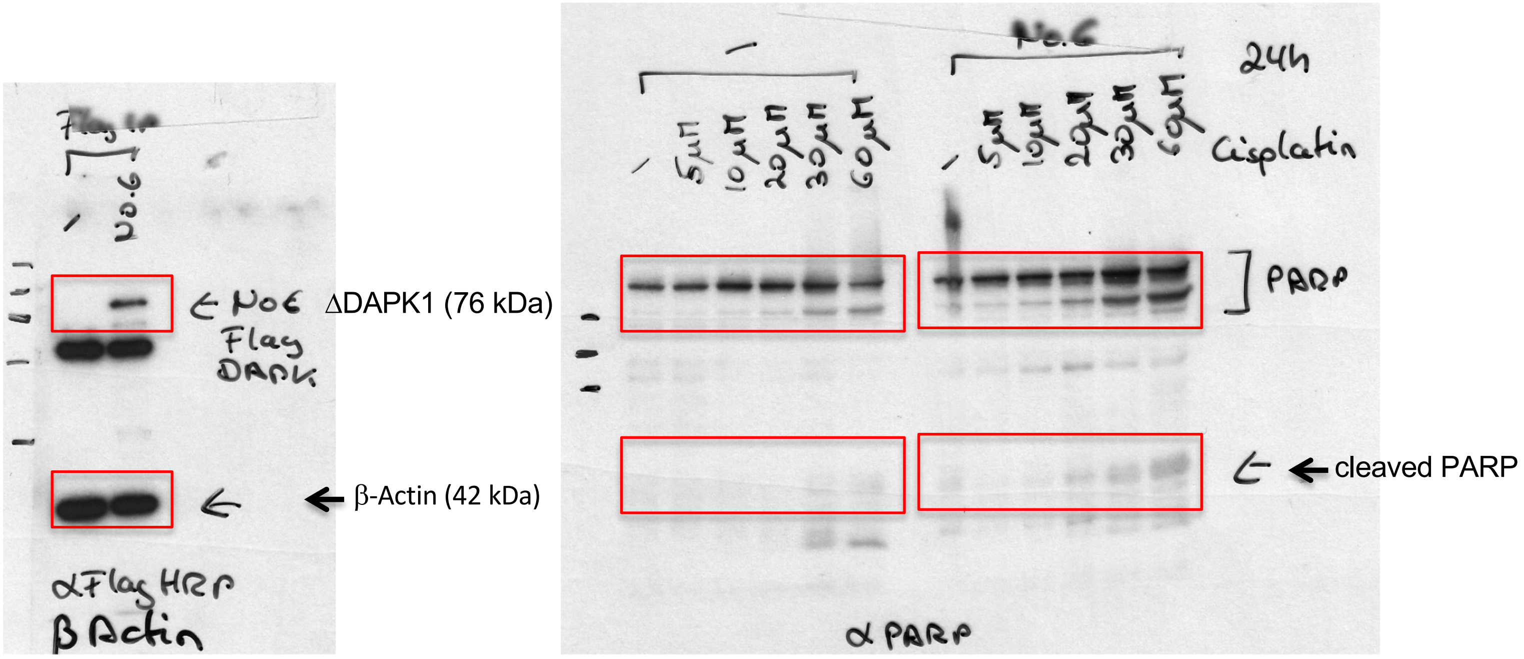

Figure S10

Figure S11

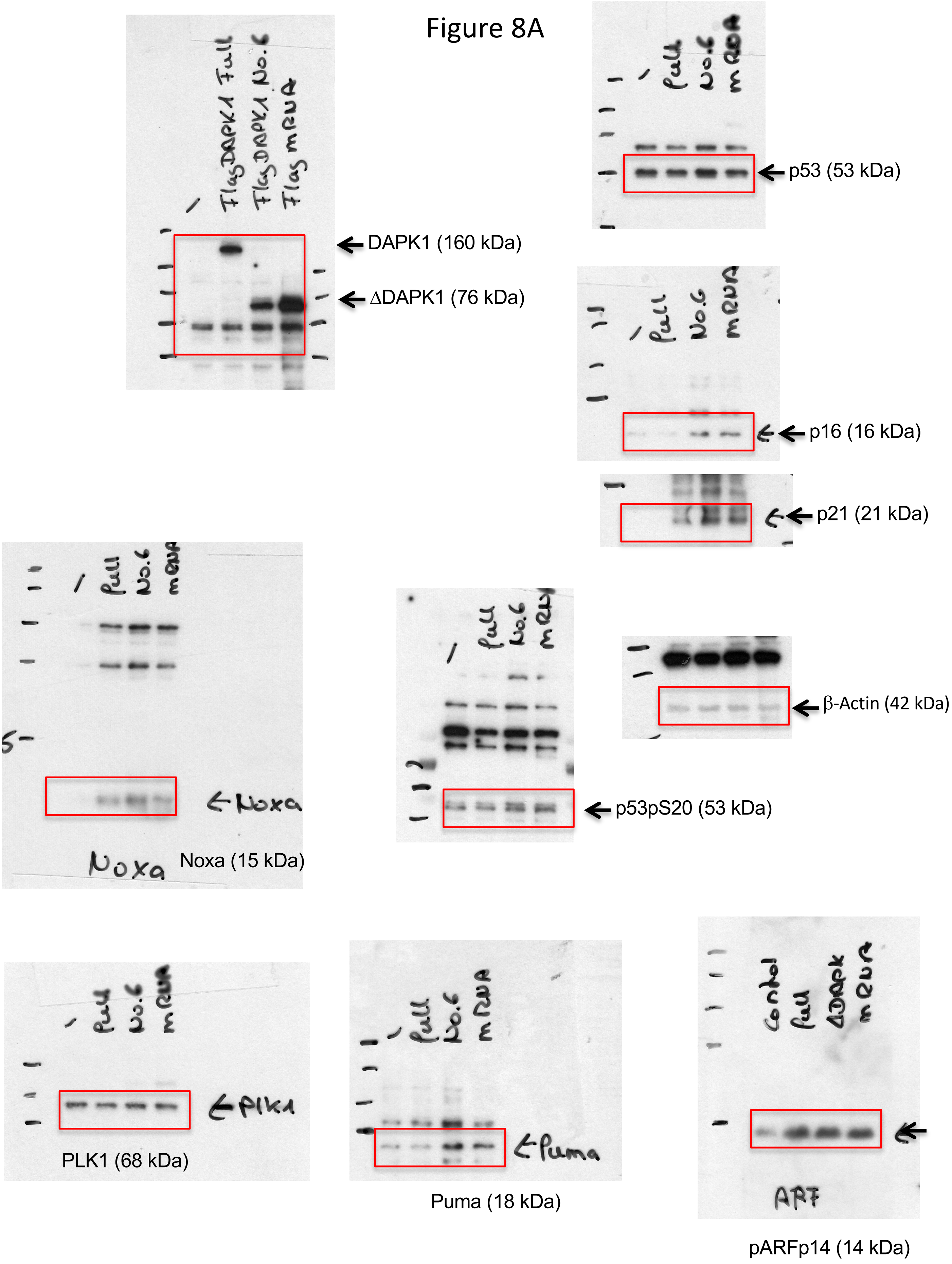

Figure 8A
Flag DAPK1 Full
Flag DAPK1 No.6
Flag mRNA
DAPK1 (160 kDa)
ΔDAPK1 (76 kDa)
Full
No.6
mRNA
p53 (53 kDa)
p16 (16 kDa)
p21 (21 kDa)
Noxa
Noxa (15 kDa)
p53pS20 (53 kDa)
β-Actin (42 kDa)
Plk1
PLK1 (68 kDa)
Puma
Puma (18 kDa)
Control
ΔDapk
ARF
pARFp14 (14 kDa)

Figure 8B

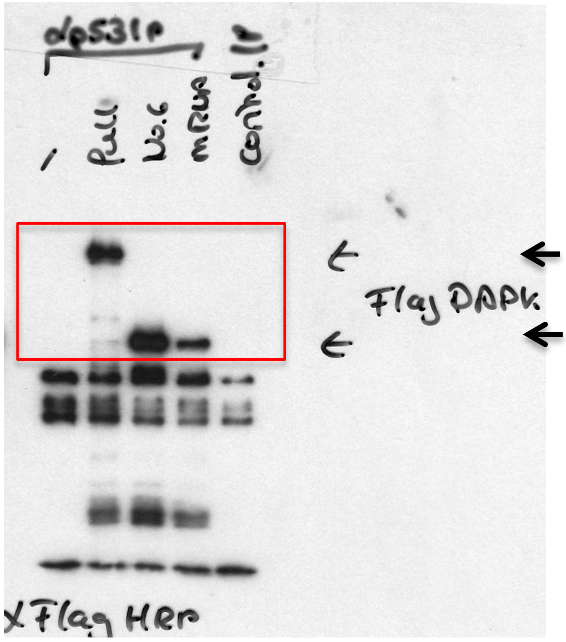

DAPK1 (160 kDa)

ΔDAPK1 (76 kDa)

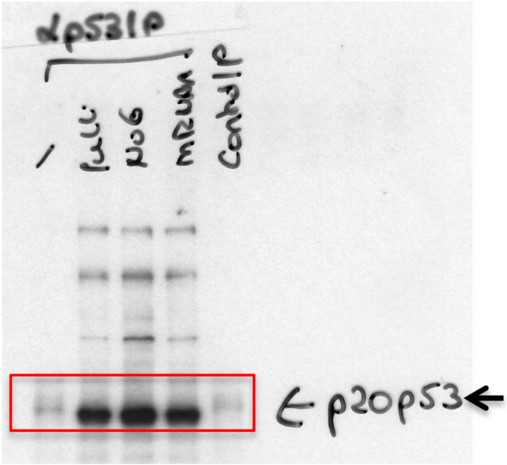

p53pS20 (53 kDa)

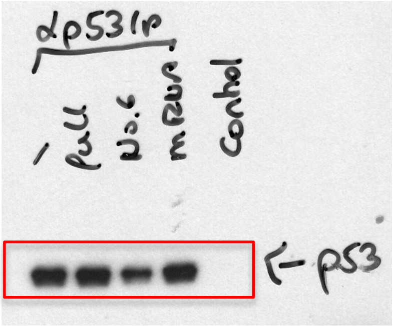

p53 (53kDa)

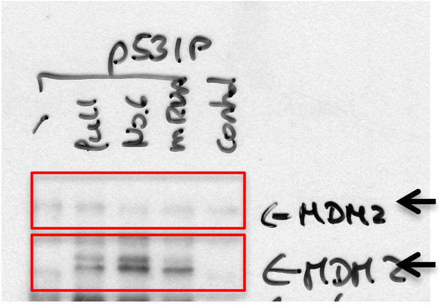

MDM2 (90 kDa)

cleaved MDM2 (60 kDa)

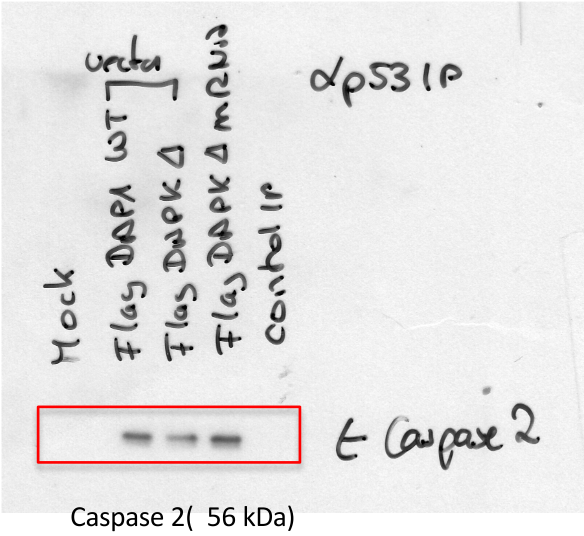

Caspase 2(56 kDa)

Figure S12

Suppl.Fig 2A

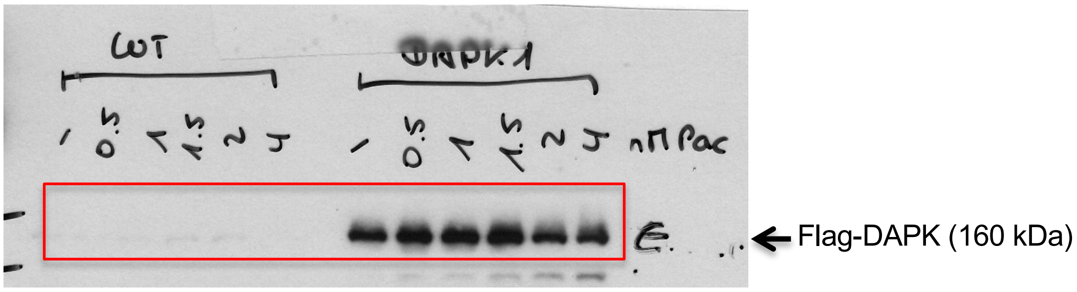

Suppl.Fig 2B

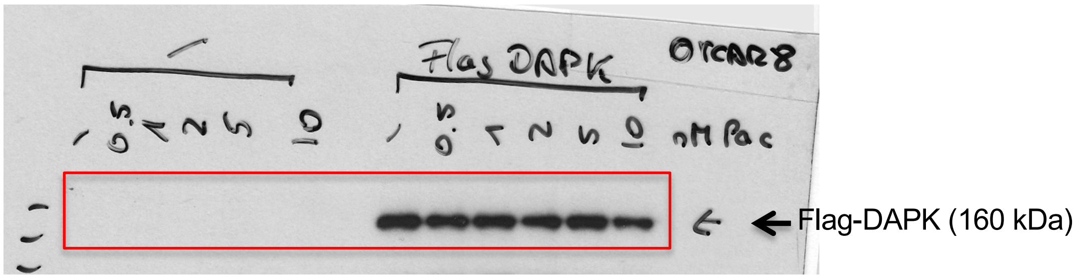

Supplement: Supplementary file 1 [file cancers-17-01910-s001.zip › cancers-3647011-supplementary.pdf]
